# Contextualized Measurement Scale Adaptation: A 4-Step Tutorial for Health Psychology Research

**DOI:** 10.3390/ijerph191912775

**Published:** 2022-10-06

**Authors:** Benjamin Ambuehl, Jennifer Inauen

**Affiliations:** 1 Eawag: Swiss Federal Institute of Aquatic Science and Technology, 8600 Duebendorf, Switzerland; 2 Institute of Psychology, University of Bern, 3012 Bern, Switzerland

**Keywords:** context specificity, measurement, scale translation and adaptation, mixed methods, psychometric properties, item response theory

## Abstract

Health psychology research is inherently context specific: Different health behaviors are executed by different target groups (e.g., gender, age) in different social structures, cultures, and environments. This asks for the adaptation of research instruments to enhance specificity. For example, when using measurement scales in new contexts, translation and psychometric validation of the instruments are necessary but not sufficient if the validity of the psychological concept behind a measurement scale has not been researched. In this study, we build on existing guidelines of translation as well as psychometric validation and present four steps on how to adapt measurement scales to a new context: Step 1 asks whether the psychological concept is found in the new context. Step 2 asks whether the measurement scale and its items are understood in the new context. Step 3 asks whether a measurement scale is valid and reliable. Step 4 asks how the items of the measurement scale perform individually. Following these four steps, measurement scales are carefully translated, adapted, and validated and can therefore be transferred to very different contexts.

## 1. Introduction

Explaining and changing health behavior is a key area of health psychology. Behavior is not only determined by the individual but is also influenced by various contextual factors [1,2] that need to be considered when measuring, explaining, and changing behavior. Measurement scales for behavior and its determinants need to guarantee an objective, reliable, and valid assessment of the measured construct [3]. When using measurement scales in new contexts (e.g., different environment, target population, or behavior), the translation and psychometric validation of the instrument are necessary but not sufficient if the validity of the psychological concept behind a measurement scale has not been researched [4]. The present paper highlights this often-overlooked issue and provides systematic guidance on how to tackle this in health psychology.

### 1.1. Context in Health Psychology

Context is the environment in which a person acts. This can be social structures [5,6], cultural scripts and behavior [7,8,9], and the physical environment [10]. Context can also change over time, for example, due to climate change [11], changes in the norms and cultural scripts, or changes over the lifespan [12]. These contextual factors influence the person, for example, by shaping attitudes or other psychosocial determinants of behavior, values, norms, and personality [13]. Measurement is context specific, too. For example, questionnaires used in the reasoned action approach include contextual factors (e.g., [14,15]) and contextual changes over time require updating measures over time as well.

Generally, context is very important to consider in research [16] and to be integrated into health psychology research for various reasons. Firstly, context can facilitate the execution of unhealthy behavior, as was found in research on habit formation [17] and smoking abstinence [18]. Secondly, context not only influences behavior directly, but context can also shape individual factors that determine behavior, such as personality traits [16], personal preferences, and response patterns [19]. Thirdly, context is measurement specific and also influences measurement, especially when using questionnaires. This is crucial because questionnaires are still one of the main methods for assessing target behavior [20], and the reliability and validity of questionnaires are key in health psychology [3]. In personality psychology, research showed that context-specific questionnaires have higher predictive validity and accuracy than general questionnaires [21]. Consequently, questionnaires need to be understood as context-specific measurement instruments. Therefore, measurement scales used in a new context, with a different population, require an adaptation of the scale and items to the new context [22]. This adaptation goes beyond mere linguistic and psychometric adaptation.

### 1.2. Scale Adaptation in Health Psychology

When established measurement scales are transferred to a different cultural context, three domains should be considered as well as focal points when adapting the survey instruments to the new context: language, culture, and measurement [23].

For language, researchers have focused on translation as an adaptation method (e.g., in medicine with the Patient-Reported Outcome Measurement Information System PROMIS [24]), referring to the linguistic translation of a scale in another language. The purpose of translation is to achieve semantic equivalence between measurement scales in both the original and new language. For example, a review [25] identifies seven elements essential to good translation of a measurement scale to a new language: (1) creating a translation team; (2) balancing literal and specific translation; (3) using back-translations; (4) testing the questionnaire in the field; (5) identifying flawed items by statistical analyses; (6) establishing new reliability, validity, and norms; and (7) contacting original source authors. Similarly, a guideline for translating psychological tests focuses solely on translation [26]. They recommend forward and backward translation and highlight that a check by a committee of experts may guarantee the accuracy of a translation. PROMIS suggests an additional cognitive debriefing interview to elicit feedback on the difficulty of items and the meaning of wording [24]. Translation and item wording, however, are not sufficient to ensure conceptual equivalence of measurement scales in different contexts [27].

For cultural adaptation, translation as adaptation methodology is extended [28] by asking participants for a cognitive debriefing or post-assessment discussion following their interviews [29]. This helps find suggestions for improving the accuracy of the translation. For cross-cultural and multicultural surveys, good practice, in turn, recommends a multistep approach (as suggested [25,30,31,32]). This includes qualitative pretesting of the translated questionnaires to ensure not only semantic (e.g., [33,34]) but additionally conceptual and content equivalence ([35,36]; used in [37]). In addition, global validation to ensure conceptual equivalence is recommended when adapting the measurement scale [26]. However, in many areas of health psychology, such as global health, this is not conducted in detail when doing cross-cultural comparative research [35].

For measurement adaptation, psychometric testing of a questionnaire is an extension to validate mathematical and statistical properties of the measurement scale [3], often through item-level analysis and subsequently refining it to improve its fit to the new context (e.g., [38,39,40,41]). Because health psychology research uses measurement scales to fulfil several empirical quality criteria [3,42], translation and validation might be sufficient for an established scale and can work to check adaptation for scales across similar cultural contexts. However, the adaptation of the measurement instruments to the specific context is often neglected or applied only in an exploratory way without actually translating or adapting the questionnaire to the new context [43,44,45]. Researchers even use measurement instruments in entirely dissimilar contexts and for very specific target behaviors without respecting the demands of extensive adaptation of the measurement instruments. This can have many reasons; one is that a convergent guideline for cross-cultural adaptation and validation of measurement protocols is lacking [46], and another is that the choice of methods depends on preference and logistics. In contrast, guidelines and reporting criteria (e.g., RECAPT [47]) have been established for adapting interventions to new contexts [48] and for the development of new measurement instruments (ABOUT toolbox [49]). However, in health psychology, to the best of the authors’ knowledge, no protocols have been published for adapting a measurement scale to a new context.

### 1.3. Measurement Scale Adaptation and Validation in New Context: A 4-Step Tutorial

To fill this gap, we developed the first comprehensive protocol for the adaptation of measurement scales to a new context. In building on three domains to be adapted [23], the tutorial comprises four steps for the successful adaptation of a measurement scale to a new context (see Table 1). We integrate and build on established protocols for conducting qualitative prestudies for measurement validation (PROMIS, [24,35,36]; used in [37]), conceptual [26] and in-detail psychometric validation to ensure measurement equivalence [3]. Compared to previous protocols, our 4-step tutorial distinguishes itself, especially by adding a step to determine whether the concept of interest can be found in the new context (Step 1). This step is crucial for validation but is often skipped in previous research. Step 2 aims to identify misunderstandings and potential problems of interpretation in the items measuring the concept found in the first step. In Step 3, the global psychometric scale performance in the new context is determined, including test–retest reliability and construct validity. Finally, Step 4 evaluates the core measurement properties and performance of items in the adapted measurement scale by using advanced psychometric analysis instruments such as item response theory (IRT). To carry out the four steps, we recommend conducting exploratory mixed-methods research, comprising a qualitative followed by a quantitative study. Table 1 summarizes the four steps, their underlying research questions and methodology.

In the following sections, we will demonstrate the steps with an empirical example. We will first describe these studies’ methods before presenting the results of the four steps. The paper concludes with a general discussion.

## 2. Empirical Example: Contextualizing the Psychological Ownership Scale to Safe Water Infrastructure in India

We exemplify the 4-step protocol in a study of psychological ownership as it relates to safe water consumption for the prevention of water-borne diseases. Psychological ownership is defined as the sense of ownership without necessarily legally owning the target of ownership [50]. Originally, psychological ownership was researched in the organizational context in the United States [51] and China [52]. Psychological ownership was later established in other areas as an important concept [53], for example, in safe water. Psychological ownership was identified as a factor related to the acceptance, use, and sustainability of safe drinking water infrastructure in various cultural contexts [54,55,56,57]. In India, researchers ([58], p. 1472) find ‘a lack of sense of ownership among local community restricting their participation in operation and maintenance of water sources. The struggle to provide the maintenance and major repairs required to keep water supply operational sustainably are well evident’. For this reason, we proposed psychological ownership as a key target when improving the functionality of safe water infrastructure in India. However, the concept stems originally from Western and organizational contexts. It has been used in other cultural contexts and for other targets, but without careful contextualization and adaptation.

As a starting point for the contextualization and adaptation to the safe water context in India, we selected the original validated measurement scale of psychological ownership [59]. There are other scales that have been used to measure psychological ownership in the context of safe water infrastructure or in the Indian context. However, they suffer from methodological shortcomings, e.g., consisting of singular items [54,55], only using parts of established psychological ownership scales with the highest face validity [56], or lacking key characteristics of the construct (e.g., possessiveness [60]).

### 2.1. Materials and Methods

We conducted an exploratory mixed-methods study [61] to adapt the psychological ownership measurement based on our 4-step protocol for contextualized measurement scale adaptation. Steps 1 and 2 were based on the qualitative part of the study, whereas Steps 3 and 4 were based on the quantitative part of the study. Ethical approval for this research was obtained from the institutional review board of the Faculty of Human Sciences of the University of Bern (Switzerland) and the Ethics Committee ICMR-Rajendra Memorial Research Institute of Medical Sciences Patna (India). Written informed consent was obtained from each participant prior to data collection.

#### 2.1.1. Qualitative Study

Interviews were conducted according to a semistructured guideline. To answer the distinct research questions of Steps 1 and 2, we followed two approaches. For Step 1, we used the grounded theory approach [62]. Grounded theory is a method for investigating the foundation of a construct [63] and is used in Step 1 to investigate participants’ understanding of the concepts, constructs, and the new context. Using the grounded theory approach validates the specific understanding of a concept in the new context because researchers do not assume the scholarly definition of a concept, but it emerges from the data bottom-up. Thus, interviews were conducted until no new information was gathered during interviews anymore. Interview guidelines can be found in Supplementary Materials S1.

In Step 2, we used think-aloud methods [64] to test the understanding of the measurement scale in the new context. Think-aloud reasoning is a method where researchers ask participants to verbalize their thoughts when choosing an answer for the items and to express their reasoning and interpretation of the choice made. This method is used frequently in health psychology to give insights into participants’ reasoning when answering questions. It helps to observe whether they understand the question as intended. Further, it helps to identify problems when answering the questions and can uncover false reasoning, misunderstandings, or doubts when answering the questions and thus validates the conceptual understanding of a questionnaire in the new context [65,66]. Interview guidelines can be found in Supplementary Materials S2.

##### Study Area and Participants

We selected villages with functional community-based piped water supply in rural Bhagalpur in the state of Bihar, India, as the study area. Community-based piped water supply was installed by government or private trusts to supply communities with drinking and cooking water free from naturally occurring arsenic. Groundwater is pumped to a central storage tank where arsenic is removed in a central filtration unit. Then, filtered water is pumped into an overhead tank and from there distributed by pipe to private or public collection taps.

Qualitative interviews were held with 18 users, non-users, and caretakers in six habitations (i.e., villages) with functional and nonfunctional safe water infrastructure.

##### Measures

Following best practice [26], the items of the original questionnaire for psychological ownership [59] were translated into Hindi by a committee of academics, working-class people, and older and younger people and double-checked by back-translating into English. If opinions diverged about a translation, it was discussed within the committee until everyone agreed on a solution. In the first phase of the interviews, no answer options were presented, as we were only interested in respondents’ reasoning. In a second phase, answer options were presented, and their interpretation was researched.

##### Analyses

For Step 1 and 2, the data were analyzed by qualitative content analysis [67]. First, the first author read four interview transcripts and inductively coded phrases and clauses as thematic elements to assemble a coding system. Second, the coding system was discussed and validated with the last author. Third, it was used to code four further interviews and, if necessary, was complemented with additional codes. Fourth, the coded interviews and all remaining interviews were coded with the more elaborate and validated coding system.

#### 2.1.2. Quantitative Study

In Steps 3 and 4, a 2-wave quantitative survey was conducted to the assess global and item-level performance of the measurement instrument in the new context. Quantitative interviews were conducted using computer-assisted personal interviewing (CAPI) methods. Additionally, we used a visual answer scale [68] to support cognitive representation and saliency of the spectrum of answer options. Interview guidelines can be found in Supplementary Materials S3.

In Step 3, psychometric properties and performance of the measurement scale were tested following criteria for scale construction [69]. In order to find out whether the quantitative measurement instrument worked well, several aspects are analyzed to characterize successful adaptation to the new context (e.g., [70]): homogeneity, internal consistency, global fit, global misfit, and overall model fit of the measurement scale. Additionally, psychometric properties, test–retest reliability, and construct validity of the adapted scale were used as quantitative indices measurement scale validity and reliability in the new context [35].

In Step 4, performance at the item level was investigated following an established protocol for scale validation in health psychology [3]. This procedure tests the applicability of the measurement scale in-depth to identify whether particular items can be improved. For example, analysis with item response theory (IRT) allows for drawing conclusions on how high discrimination of items is and how informative the scale is to distinguish between respondents with different traits and thus validates the measurement scale and its items in the new context [71].

##### Study Area and Participants

As study areas, we selected four villages with functional community-based piped water supply in rural Bhagalpur in the State of Bihar, India. Community-based piped water supply was installed by government or private trusts to supply communities with arsenic-free drinking and cooking water.

We aimed to select 30 households per village randomly. With the exception of one village, the caretakers of the infrastructure were also interviewed. The first survey wave was conducted in March 2019, followed by a 6-month time lag that included a monsoon, followed by the second survey wave in September. Quantitative data was collected from a total of *N* = 193 participants, who categorized themselves as using the safe water infrastructure (*n* =111), not using the infrastructure, or using other water source as main source (*n* = 79), or as being the caretaker of the safe water infrastructure (*n* = 3).

##### Measures

In addition to psychological ownership, water collection practices and several theory-based psychosocial determinants were assessed because they were found to be related to psychological ownership in previous studies. The questionnaire can be found in Supplementary Materials S3.

Psychological ownership. In Steps 1 and 2, we used an adapted individual psychological ownership scale [59] to assess psychological ownership of the water system in the Indian context (see Table 2).

Routes to psychological ownership. Three routes are established on how the sense of ownership evokes [51]: by having control over the target of ownership, by being familiar with and having intimate knowledge about the target of ownership, and by investing the self into the target of ownership. These three routes were measured with multiple items each.

Water collection practices. As explained in the introduction, psychological ownership was found to have several effects on people’s water collection practices. Some of the most important ones were included in this survey: use [54], habit [72], acceptance for infrastructure [54], and commitment to caretaking [58].

##### Analyses

To quantitatively validate the psychological ownership scale, we examined the homogeneity as internal consistency of the ownership items. Subsequently, we tested the unidimensionality (corresponding with the finding of [59] of the measurement scale with confirmatory factor analysis (CFA) using the lavaan package in R [73]). CFA tests a hypothesized structure with model fit statistics and parameter estimates [74]. Model fit indices need to be judged against recommended thresholds: the Tucker–Lewis index (TLI) and the comparative fit index (CFI) > 0.95; root mean square error of approximation (RMSEA) < 0.06; and χ2 *p* value > 0.05 [75,76]. To assess discriminant and criterion validity, we performed simple regression analyses for routes and continuous outcomes of psychological ownership. Logistic regression analyses were conducted for dichotomous outcomes. Our sample size did not allow a nested data structure for generalized estimating equations (GEE) models.

Homogeneity and IRT were calculated using the mokken package in R [77] and the eRm package in R [78]. For a detailed description of this analysis protocol, discussion, and interpretation of the results, we refer to [3].

### 2.2. Results

#### 2.2.1. Step 1: Is the Psychological Concept Found in the New Context?

In our example, Step 1 investigates the understanding of the concept of psychological ownership for safe water infrastructure in Bihar, India and answers research question 1: How is psychological ownership understood in Bihar, India? The results of the qualitative interviews to answer this question are presented as follows, structured by the understanding of the construct itself, its antecedents, and its consequences.

##### Psychological Ownership and Dimensionality of Constructs

Villagers had several understandings of individual psychological ownership. They explained that one dimension of the translation of psychological ownership is ‘possession,’ whereas the second dimension is ‘controlling’:

‘Ownership has two translations in Hindi: leadership/being the head = *svaamitv* and property/possession = *malikh*’

However, they explained that both translations have one meaning in common:

‘I think that these are like personal things.’

Additionally, this personal belonging was also seen by others in the community; it had a third, social dimension:

‘It is known to everyone here that this cow belongs to me. The whole community here knows that this cow is mine, so they will say accordingly.’

Moreover, as a third dimension, psychological ownership was also understood as an instrument of power:

‘This feels like having the power over these things, having the full amount of control.’

Villagers also distinguished individual psychological ownership and collective psychological ownership, depending on how the target of ownership was created and who invested in it:

‘The temple is for everyone, but this filter must belong to the person who gave their land for its installation.’

Moreover, by who the target of ownership benefits or how the target of ownership can be used:

[Collective ownership is when] ‘their use can be shared.’

They also causally linked the routes to (investment) and consequences of (use) collective psychological ownership:

‘Everyone from the community can use it as they have contributed equally.’

However, the collectiveness was still sharply defined:

‘This temple is for the whole community as they have contributed to its construction. Well only for the Hindu community.’

Collective psychological ownership was seen as a very elaborate and holistic perspective on how the community was responsible for the target of ownership:

[Collective psychological ownership depends on] ‘mutual consent by the community and the whole community should be able to pay or contribute significantly to that. We may do our own boring then and subsequently will have our own connectivity. The caretaker will be from us and he/she will be responsible to look after the unit.’

##### Antecedents to Psychological Ownership

When interviewing villagers about the antecedents of their feeling of ownership, an answer mentioned very frequently was about being the head or leader of something:

‘When something is in his hands, he then is the head of something and therefore has the feeling of owning something.’

Or they reported having invested tokens, money, or labor in installation and maintenance:

[It is ours, because…]

‘We have spent Rs. 10,000 in its installation with the help of some labors having a depth of 50 feet. The labor cost Rs. 2000 each.’

[It is ours, because…]

‘Ownership is not simply equal to costs, it is more that guarding leads to ownership.’

[It is ours, because…]

‘We have donated this land for the installation of the unit.’

[It is ours, because…]

‘If I am looking after the house and cattle, it is mine then.’

##### Consequences of Psychological Ownership

When reporting a sense of ownership, villagers referred to their unlimited use of the target of ownership:

‘Only for me and related to mine and not to the others, there are no restrictions in use.’

Moreover, that a sense of ownership structures social interactions and influences the behavior of individuals in the community:

‘We did not prefer to go there because that source is someone else’s and we don’t like going there.’

However, ownership was found to have implications:

‘No direct benefits, it depends on functionality (if there is benefit possible, owning is good) […] I have to look after everything...’

More in detail, it also involves a duty to look after the target of ownership and a feeling of responsibility and accountability in the community:

‘And the caretaking of the things is still a duty of the owner. So he feels a certain responsibility for the things as well as no limits in using them. Considering other people using his things, he has a bit of an unsafe feeling.’

In summary, the consequences of psychological ownership were understood as feelings of power and control over the target of ownership:

‘This feels like having the power over these things, having the full amount of control, like being a leader.’

##### Conclusions on Step 1: The Concept Is Relevant and Understood in New Context

In Step 1, we found the concept of psychological ownership to be relevant and understandable in the context of safe water supply and collection in India. However, we found that psychological ownership in Bihar was understood as a multidimensional construct with the additional dimensions of possession, social acknowledgement, and power. In the literature, new scales developed to assess psychological ownership are often multidimensional [60,79,80]. This was not the case in the original context [59,81]. Even so, in our data, we found the original dimension, possession, to be present. Antecedents, consequences, and differentiation between individual and collective psychological ownership were found, which aligned with previous findings [53,82].

#### 2.2.2. Step 2: Are the Measurement Scale and Its Items Understood?

In Step 2, we tested whether the items of the psychological ownership scale are understood in the new context: safe water infrastructure in Bihar, India. Based on the results of Step 1, we developed additional items to measure psychological ownership three-dimensionally. However, when testing for homogeneity of the measurement scale and criterion validity in Step 2, we did not identify a better model when including a three-dimensional measurement scale. Overall fit indices of the three-dimensional measurement scale can be found in Supplementary Materials S4: the three-dimensional scale did not fit the data well. Because of this and because qualitative findings of Step 1 converge on the ‘possession’ as a central dimension, we continue this protocol by reporting only on the measurement scale of the ‘possessiveness’ dimension.

##### Introduction to the Scale: Examples for the Psychological Construct

When asking about examples of having the sense of ownership, villagers mentioned the following:

‘I have this house, my vehicles and cattle, my agricultural land.’

‘He owns the house, land, cows, a bike.’

‘He owns cows (by buying), land, clothes, mobile, shoes, tractor, TV, motorbikes, knowledge, handpump.’

‘I have my house, my shop, my garden and agricultural land and one bicycle.’

‘I am the head of my family.’

Additionally, we asked about how they call the community’s organization of the water supply, and participants responded in agreement:

‘Water scheme of the community. And privately, I also have a handpump.’

According to these results, we rephrased the introduction to the psychological ownership scale and reworded the target of ownership in the items (Table 2).

##### Items That Worked as Intended

When responding to the items by thinking aloud, respondents that perceived only community ownership for the safe water infrastructure agreed, for example, on the item ‘This is our water scheme.’:

‘Yes, it is true.’

They also agreed on the item, ‘Most of the people that live in this village feel as though they own the water scheme.’:

‘Yes. And the persons who want this water they had the talk with the caretaker. The person who wants this water can take this water.’

‘Yes, we feel so. Water is life after all. We would like to have the same filter scheme with HOUSEHOLD piped water from river Ganga that gives us hope in the beginning for safe drinking water.’

‘Yes it’s for community—it is for the wellbeing of communities.’

Consistent with their reasoning that there is no personal ownership, they denied personal ownership when responding to the item ‘This is my water scheme.’:

‘No, it is not true.’

Neither they agreed on the item ‘I feel a very high degree of ownership for this water scheme.’

‘No, I don’t think so.’

For others, personal and community ownership can also go together. They responded to the item ‘This is my water scheme’ the following:

‘Yes, it is mine, as well as for others, as long as it is giving the water to the community. It’s never in my mind that this system is entirely mine.’

To the item ‘This is our community water scheme.’ they answered the following:

‘I’m not saying that it is my personal unit, this is for whole community.’

And to the item ‘Most of the people that live in this village feel as though they own the water scheme.’ the following:

‘Yes, they think like that. They have given their signature on the consent form in front of the Government officials.’

##### Item That Caused Confusion and Was Not Understood

The inversely stated item ‘It is hard for me to think about this water scheme as mine’ caused problems when answering. Many respondents were confused:

‘I don’t understand. Because the filter is my own and the water belongs to everyone, because it makes them healthy.’

Or they did not understand what was being asked:

‘I don’t understand. If I want to get ownership, I am willing to pay for owning.’

##### Conclusions on Step 2: Most Items Are Understood

The adapted questionnaire was tested and understood by the villagers. The transfer from the nontangible original target of an organization to the tangible one of infrastructure was found to be easily possible, and the corresponding terminology was found for the new target of ownership. Think-aloud reasoning about examples for psychological ownership highlighted which adaptations were necessary. By changing the introduction and examples provided according to the results of this step, we adapted the measurement scale minimally but still precisely for the new cultural context and target of ownership. Additionally, think-aloud responses also hinted at difficulties when the inversely stated item was misunderstood or not understood at all.

Previous studies (e.g., [60]) established new measurement scales and rejected the original scale. Often, they expanded the conceptualization of the original scale and, in turn, developed a new scale measuring psychological ownership as a multidimensional scale (as the data structure indicated). However, when adapting the original single-factorial scale, we found such expansion to be unnecessary. As a result of this qualitative step, wording and examples in the introduction were adapted, and the measurement scale was cleared for pretesting in a quantitative study.

#### 2.2.3. Step 3: Is the Measurement Scale Valid and Reliable in the New Context?

In our example, Step 3 investigates the global structure and properties of the measurement scale of psychological ownership for safe water infrastructure in Bihar, India and answers research question 3: Is the Measurement Scale Valid and Reliable in the New Context?

The original seven-item scale showed a significant chi-square (χ2 = 101.167, 14 d.f., *p* < 0.05), high RMSEA = 0.138, and low CFI = 0.871. Completely standardized factor loadings ranged from −0.017 to 0.784.

In the first iteration, we deleted two items (PO_017 and PO_020) with the lowest item-total correlation. The shortened 5-item model showed still a significant chi-square (χ2 = 69.532, 5 d.f., *p* < 0.05), high RMSEA = 0.198, and higher CFI = 0.899). Completely standardized factor loadings ranged from 0.592 to 0.780.

**Figure 1:**
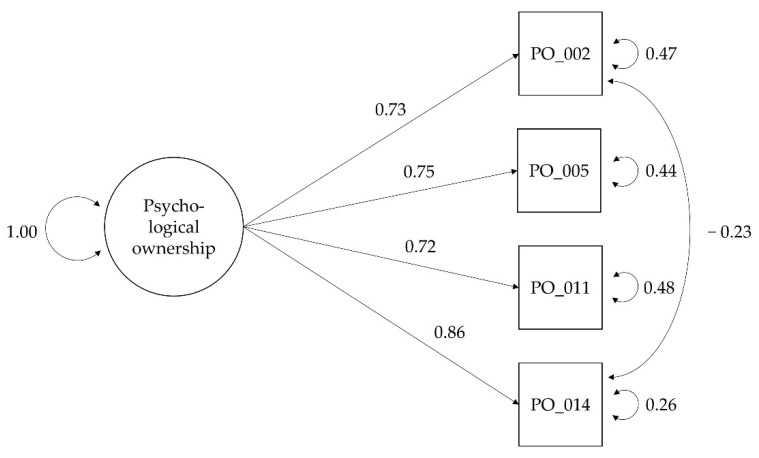
CFA model for 4-item measurement scale. Note. 4-item psychological ownership measurement scale: a one-factor model that allows for covariances between items with similar wording (PO_002 and PO_014: ‘This is ______ water scheme.’).

In the second iteration, we deleted one item with the lowest factor loading and allowed for covariances between items with similar wording. The shortened 4-item model (Figure 1) showed good fit indices: a nonsignificant chi-square (χ2 = 2.779, 1 d.f., *p* = 0.351), low RMSEA = 0.074 [0.000; 0.183], and high CFI = 0.996. Completely standardized factor loadings ranged from 0.721 to 0.857 (Figure 1). With *N* = 193, we found internal consistency (Cronbach’s α) to be high at both the first measurement time point (Cronbach’s α1 = 0.826) and at the second measurement time point (Cronbach’s α2 = 0.881). Test–retest reliability as ICC [83] was moderate: 0.64 [0.33; 0.81].

**Figure 2:**
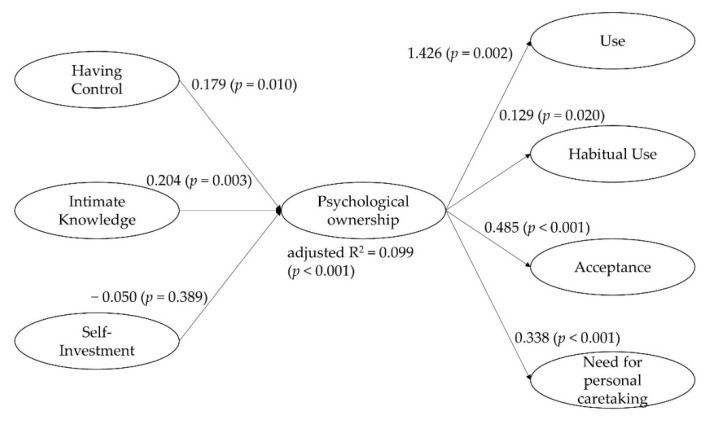
Criterion validity of the psychological ownership scale. Note. A summary of multiple linear regressions and a logistic regression (use = 1; nonuse = 0). *N* = 328. Highlighted with grey background, having control and intimate knowledge related significantly to psychological ownership such as routes, whereas self-investment did not. Psychological ownership was related to the use of the water scheme, habitual use, its acceptance, and the perceived need to take care of the water scheme.

We found that the criterion validity of psychological ownership differed between users and non-users of the water scheme and was correlated to certain routes and consequences (Figure 2). Routes and consequences were defined and conceptualized a priori from theory.

##### Conclusions from Step 3: A Contextualized 4-Item Version Shows Good Psychometric Properties

The results of this step showed that the contextualized and translated psychological ownership scale by [59] could be transferred to the safe water infrastructure context in India. After several iterations, good overall fit indices were found for a 4-item scale. The results from the stepwise exclusion of items converge with the qualitative findings in Step 2, where the inversely stated item was not understood. Internal consistency was increased when this item was deleted from the scale.

The shortened 4-item scale showed comparable fit indices to those of its original context (*N* = 227; internal consistency: α1 = 0.93, α2 = 0.89; χ2: 3.74 (df = 2, *p* > 0.05); RMSEA: 0.06; CFI: 0.99; factor loadings: 0.73–0.93; [59]) apart from test–retest reliability, which was slightly lower in our study than in the original context (test–retest reliability: 3-month lag, r = 0.72). However, there are plausible explanations for this. Firstly, we tested the measurement scale in an unstable environment; the monsoon changes people’s water collection practices, as they switch to water collection sources that are not flooded. Second, the test–retest lag was about 6 months, compared to 3 months in the original context. Therefore, we suggest that test–retest reliability be assessed again over a shorter time interval during the dry season.

We found good criterion validity, differing between the consequences of psychological ownership of the water scheme. Furthermore, we also found different theorized routes to be associated with psychological ownership.

#### 2.2.4. Step 4: How do the Items Perform Individually in the New Context?

After overall criteria, we aim now to analyse performance of the measurement scale in-depth on item level. In our example, Step 4 tests the applicability of the measurement scale items to identify whether particular items can be improved and how they discriminate between respondents.

##### Descriptive Statistics

Descriptive analyses of all items included in the measurement scale are displayed in Table 3. Interitem correlation (Table 4) shows very low correlations of items PO_017 and PO_020 with the other items.

**Figure 3:**
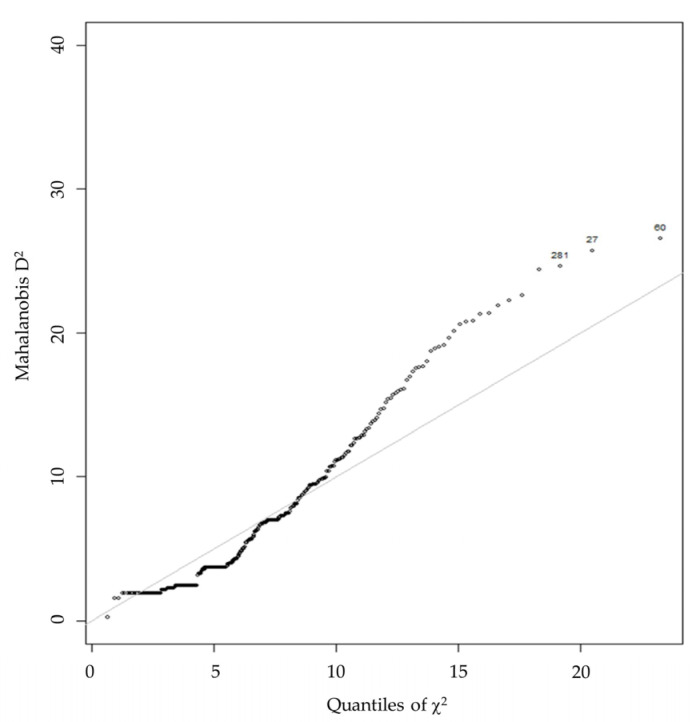
Multivariate outliers in item set. Note. This plot of Mahalanobis D2 vs. quantiles of Chi2 shows an upward bending on the left side and a downward bending on the right side. This indicates possible outliers at the top end. However, as they are not found to be extremely unlikely, they were left in the sample size.

Analysis for multivariate outliers by plotting a Mahalanobis D2 (Figure 3) found no unusually responding participants that would warrant exclusion.

##### Nonparametric Item Response Theory (IRT)

Homogeneity, indicating whether the items are scalable and measuring the same construct as the scale, is displayed in Table 5. For two items (PO_017 & PO_020), homogeneity was below 0.3, indicating problematic item performance.

**Figure 4:**
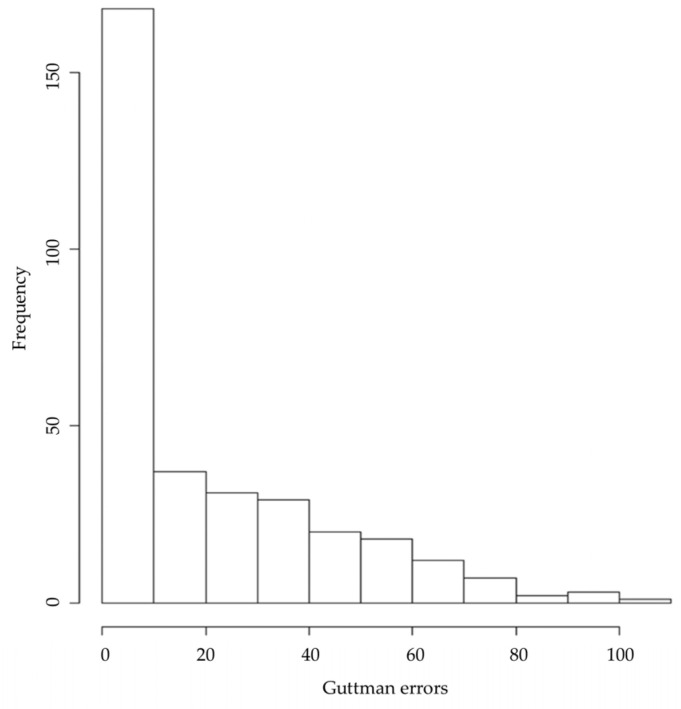
Histogram of Guttman errors for all item set. Note. Evaluation of Guttman errors of every item for participants. This evaluation flags aberrant response data. Counting the number of Guttman errors is an alternative to more complex statistics for determining nonfitting item score patterns [84]. Low numbers of Guttman errors are therefore a sign of well performing items.

In turn, analysis of the person-fit (by distribution of Guttman errors) is shown in Figure 4.

In order to test unidimensionality, the results of an automated item selection procedure (AISP) with all items are shown in Table 6. Cell values of 0 indicate poor item performance and items classified as unscalable (items PO_017 & PO_020).

Based on the assumption that latent variables are interval variables, monotonicity assesses whether item difficulty increases for every item. Monotonicity is shown in Table 7. It highlights that none of the items has a critical value (crit) > 0.8 that would warrant exclusion.

In IRT, easy items are usually presented at the beginning, and items gradually become more difficult. Invariant item ordering (IIO) assesses whether items retain the same order of difficulty over all levels of the latent variable. Table 8 shows that some items have very high critical values. One by one, each item with the highest value is excluded. After item PO_008 has been excluded, the IIO is displayed in Table 9.

##### Parametric Item Response Theory (IRT)

As our scale and items did not measure all levels of the latent continuum proportionally, we decided to run nonparametric IRT in Step 4.2, and thus Step 4.3 was not conducted.

##### Factor Analysis

See Step 3 above.

##### Classical Test Theory

**Figure 5:**
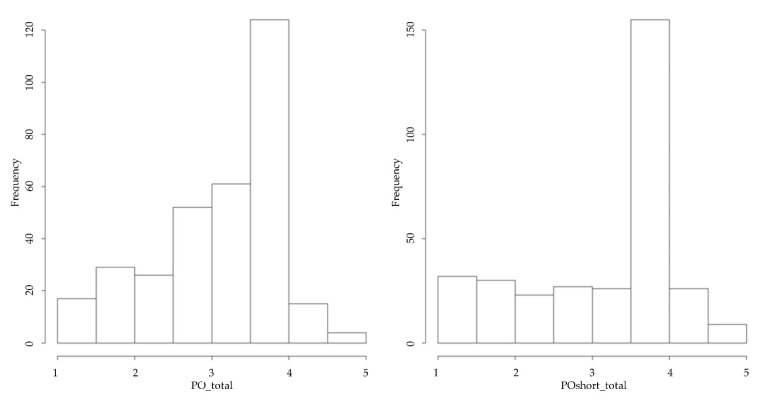
Histogram of original scale (left) and shortened scale (right) at measurement time point 1. Note. Correlation of the two scales is *r* = 0.84. Abbreviations: PO_total = original seven-item scale measuring psychological ownership; POshort_total = shortened 4-item scale measuring psychological ownership.

Classical test theory includes a variety of analyses. A range of indicators of internal consistency and reliability are displayed in Table 9. Descriptive statistics of a shorter scale than the original are reported in Table 10 and Table 11, and the histogram comparing the variance of the two is displayed in Figure 5.

##### Conclusions from Step 4: The Results Confirm the 4-Item Scale

In-depth psychometric analysis at the item level converged with findings from Steps 2 and 3, where we already identified one question as performing poorly: the inversely stated item caused reasonable problems for respondents and did not fit the CFA model. Inter-item correlation showed that one particular item, PO_017, did not correlate with the other items of the measurement scale. Additionally, homogeneity analyses revealed that item PO_017 did not have scalable properties. Overall, these analyses corroborated previous findings and therefore strongly suggest the need to exclude this item from the measurement scale. In invariant item ordering, two further items were identified as not meeting the criteria for monotonicity. Thus, to achieve a scale measuring a single construct and so that differences between respondents are appropriately represented in their sum and average scores, two items were excluded from the scale, resulting in a definitive scale of four items measuring psychological ownership.

## 3. General Discussion

In this article, we introduced a comprehensive 4-step protocol to adapt psychological measurement instruments to a new context. The protocol was based on previous recommendations on scale adaptation but extended by adding a first step where the concept itself is validated prior to scale validation. The example investigation of the concept of psychological ownership and its measurement scale illustrates that it is not always necessary to develop a measurement instrument in a new context from scratch; instead, careful adaptation and validation procedures allow existing measurement scales to be transferred successfully to new contexts. In Step 1 of our protocol, using the grounded theory approach, we confirmed the understanding and relevance of the construct in the new context and revealed examples with which to frame the introduction to the questionnaire (Step 1). In Step 2, think-aloud interviews helped identify respondents’ difficulties and reasoning processes when answering the questions of the psychological ownership scale. Then, the quantitative analyses of the psychometric properties at scale and item levels in Steps 3 and 4 confirmed the validity and reliability of the scale in the new context. The comparison of the qualitative and quantitative findings also showed convergence: difficulties reported when understanding or answering items in the think-aloud paradigm aligned with impaired psychometric properties of the same items. This provides strong evidence that such items have to be excluded from the measurement scale to ensure that the measurement instrument is valid and reliable.

Previous adaptation protocols predominantly focused on translation. Our 4-step protocol significantly expands adaptation practices to reflect the three domains recommended to be considered when adapting a measurement instrument to a new context: culture, language, and measurement [23]. In particular, our protocol combines an initial qualitative investigation of the concept behind the measurement scale with an in-depth quantitative psychometric analysis of the scale and items.

We targeted culture in qualitative Step 1: we adapted the introduction by changing the examples of psychological ownership and by identifying whether the concept was understood in the new context. Perhaps cultural differences in other cases require more profound adaptation, and thus integration of qualitative and quantitative findings may cause problems [85]. We targeted language in qualitative Step 2: we adapted comprehensibility and language by using words and sentence structures corresponding to the local language [86]. This pragmatic-driven adaptation recognizes that language is used in a social context. Lastly, in measurement, we adapted the questions’ familiarity by introducing the question format with an example. We adapted the question format by introducing a two-step sequential question style and by providing a visual answer scale.

Qualitative and quantitative approaches combined present a robust approach to adapting measurement instruments to new contexts where conceptual understanding is potentially different. In these instances, validating the concept in the new context is of particular importance as translation only (e.g., PROMIS) cannot guarantee conceptual equivalence of the measurement instrument. However, such a mixed-methods approach to conceptual adaptation is only appropriate when the context substantially differs because it is a resource- and time-intensive process. It may not be worthwhile when measurement instruments are adapted to new contexts or targets that are similar or closely related to the originals. If no conceptual qualitative investigation of the topic is necessary, we suggest at least using think-aloud methods as described in Step 2 and validating the questionnaire with Steps 3 and 4.

It is a common process to shape a measurement scale when pilot testing it. This increases its efficiency, validity, and reliability in future applications. However, it needs to be done carefully to avoid impairing criterion validity [87]. In our protocol, evidence from qualitative and quantitative assessments and global and item-level assessments are used, and adjustments to the measurement scale are only made if the findings converge. Furthermore, sampling strategies to include a broad range of participants with different perceptions of the concept and different contexts is one aspect of preventing biases because of measurement invariance [88]. For example, we included participants from various groups (users and non-users, different socio-economic levels, religions, and castes) and diverse occasions in our sampling (e.g., functional and broken infrastructure) to account for the different understandings and situations relevant to our construct [88]. Ideally, an in-depth analysis of measurement invariance (e.g., over time) is something that should be done in addition to this protocol.

The main limitation of this mixed-methods approach is the amount of work required to adapt a scale to a new context. As the example showed, an entire prestudy is needed for the first adaptation of a scale to a new context. However, Step 4 showed that such a protocol is only worthwhile when a scale of several items is adapted and tested, as several items may not match the criteria and need to be excluded from producing a well-functioning measurement scale. Nevertheless, we advocate the necessity of such extensive adaptation procedures, as only through the qualitative steps can reliable and valid measurement scales of psychological concepts be guaranteed to achieve coherence of measurement instruments across contexts and fit specific contexts at the same time. Furthermore, contextual adaptation frameworks may even be used to adapt intervention protocols. It is particularly important that intervention activities are tailored to their contexts because precise mechanisms of action are necessary to unfold effectiveness [89]. Such tailoring can also be achieved by conducting qualitative steps to assess the relevance [90], difficulties, and understandability of intervention activities, for example, in a person-based approach [91]. We conclude from following this 4-step adaptation approach that carefully translated, adapted, and validated psychological questionnaires can be transferred to very different contexts.

## Figures and Tables

**Table 1:** 4-step protocol for contextualized measurement scale adaptation.

Step	Research Question	Method	Alternative Procedure
1	Is the psychological concept found?	Qualitative interviews using grounded-theory approach.	If concept is not understood or it has different dimensions: develop new items for potentially measuring these new understandings and dimensions.
2	Are the measurement scale and its items understood?	Qualitative interviews using think aloud approach.	If items are not understood, reasoning does not match intended conceptualization: exclude items from measurement scale.
3	Is the measurement scale valid and reliable?	Quantitative survey with minimum two survey waves.	If items do not fit in factor analysis; criterion validity; internal consistency; test–retest reliability: exclude items from scale.
4	How do the items perform individually?	Quantitative survey with minimum two survey waves.	See [3].

Note. 4-step protocol summary with research questions for every step and proposed method to answer research question.

**Table 2:** Psychological ownership measurement scale.

	Original Scale [59]	Adapted Scale
Introduction	Think about the home or the boat or the cabin that you own and the experiences and feelings associated with the statement ‘this is my (our) house!’. The following questions deal with the ‘sense of ownership’ that you feel for the organization that you work for. Indicate the degree to which you personally agree or disagree with the following statements:	Think about the home or *the cow or the bike or the mobile* that you own and the experiences and feelings associated with the statement *‘this is my home!’, ‘this is my cow!’, ‘this is my bike!’, ‘this is my mobile!’*. The following questions deal with the ‘sense of ownership’ that you feel for the *water scheme* that you *have in this community*. Indicate the degree to which you personally agree or disagree with the following statements:
PO_002 *	This is my organization.	This is my *water scheme*.
PO_005 *	I sense that this organization is our company.	I sense that this *water scheme* is our *water scheme*.
PO_008	I feel a very high degree of personal ownership for this organization.	I feel a very high degree of personal ownership for this *water scheme*.
PO_011 *	I sense that this is my company.	I sense that this is my *water scheme*.
PO_014 *	This is our company.	This is our *water scheme*.
PO_017	It is hard for me to think about this organization as mine.	It is hard for me to think about this organization *water scheme* as mine.
PO_020	Most of the people that feel as though they own the company.	Most of the people that *live in this village* feel as though they own the *water scheme*.

Note. Strikethrough font: original wording of scale (Context: Western; Target of ownership: organization) [59]. Italic font: Adapted wording of scale (Context: Indian; Target of ownership: community water supply scheme) used in Step 2. * These items form the definitive adapted measurement scale, after the entire adaptation and validation procedure.

**Table 3:** Descriptive statistics of all items.

	*M*	*SD*	*f*	*f%*	Coding
Psychological Ownership for the Water System at Baseline					
This is my water scheme. (PO_002)	3.45	1.26	328	100	1–5
I strongly disagree.			49	14.94	1
I disagree a little.			33	10.06	2
I neither agree nor disagree.			5	1.52	3
I agree a little.			204	62.20	4
I strongly agree.			37	11.28	5
I sense that this water scheme is our water scheme. (PO_005)	3.38	1.26	328	100	1–5
I strongly disagree.			52	15.85	1
I disagree a little.			34	10.37	2
I neither agree nor disagree.			13	3.96	3
I agree a little.			197	60.06	4
I strongly agree.			32	9.76	5
I feel a very high degree of personal ownership for this water scheme. (PO_008)	3.06	1.42	328	100	1–5
I strongly disagree.			85	25.91	1
I disagree a little.			31	9.45	2
I neither agree nor disagree.			25	7.62	3
I agree a little.			152	46.34	4
I strongly agree.			35	10.67	5
I sense that this is my water scheme. (PO_011)	3.17	1.34	328	100	1–5
I strongly disagree.			70	21.34	1
I disagree a little.			32	9.76	2
I neither agree nor disagree.			25	7.62	3
I agree a little.			174	53.05	4
I strongly agree.			27	8.23	5
This is our water scheme. (PO_014)	3.31	1.3	328	100	1–5
I strongly disagree.			55	16.77	1
I disagree a little.			41	12.50	2
I neither agree nor disagree.			15	4.57	3
I agree a little.			182	55.49	4
I strongly agree.			35	10.67	5
It is hard for me to think about this water scheme as mine. (PO_017)	3.09	0.97	328	100	1–5
I strongly disagree.			0	0.00	5
I disagree a little.			139	42.38	4
I neither agree nor disagree.			20	6.10	3
I agree a little.			169	51.52	2
I strongly agree.			0	0.00	1
Most of the people that live in this village feel as though they own the water scheme. (PO_020)	2.71	1.5	328	100	1–5
I strongly disagree.			117	35.67	1
I disagree a little.			43	13.11	2
I neither agree nor disagree.			25	7.62	3
I agree a little.			105	32.01	4
I strongly agree.			38	11.59	5

Note: *N* = 328. *f* = absolute frequency. *f%* = relative frequency. *M* = Mean; *SD* = Standard deviation.

**Table 4:** Interitem correlations.

	PO_002	PO_005	PO_008	PO_011	PO_014	PO_017	PO_020
PO_002	1.00						
PO_005	0.50	1.00					
PO_008	0.44	0.33	1.00				
PO_011	0.43	0.49	0.46	1.00			
PO_014	0.34	0.56	0.28	0.61	1.00		
PO_017	0.12	−0.07	0.05	−0.02	−0.03	1.00	
PO_020	0.10	0.14	0.27	0.20	0.11	0.06	1.00

Note: N = 328. Spearman correlations. Abbreviations: none.

**Table 5:** Homogeneity values of all items.

	*Scale H*	*SE*	*Item H*	*SE*
**Psychological ownership for the water system at baseline**	0.34	0.03		
This is my water scheme. (PO_002)			0.44	−0.04
I sense that this water scheme is our water scheme. (PO_005)			0.43	−0.04
I feel a very high degree of personal ownership for this water scheme. (PO_008)			0.39	−0.04
I sense that this is my water scheme. (PO_011)			0.45	−0.03
This is our water scheme. (PO_014)			0.39	−0.04
It is hard for me to think about this water scheme as mine. (PO_017)			0.01	−0.05
Most of the people that live in this village feel as though they own the water scheme. (PO_020)			0.19	−0.04

Note. *N* = 328. The complete item set has a homogeneity value *H*(*SE*) = 0.34. (0.03). None of the items failed to meet the local independence criterion; *SE* = standard error.

**Table 6:** Automated item selection procedure (AISP) for increasing homogeneity (H) thresholds (c).

Item	Homogeneity Threshold Levels											
	c = 0.05	c = 0.10	c = 0.15	c = 0.20	c = 0.25	c = 0.30	c = 0.35	c = 0.40	c = 0.45	c = 0.50	c = 0.55	c = 0.60
This is my water scheme. (PO_002)	1	1	1	1	1	1	1	1	1	1	2	0
I sense that this water scheme is our water scheme. (PO_005)	1	1	1	1	1	1	1	1	1	1	1	1
I feel a very high degree of personal ownership for this water scheme. (PO_008)	1	1	1	1	1	1	1	1	1	0	2	0
I sense that this is my water scheme. (PO_011)	1	1	1	1	1	1	1	1	1	1	1	1
This is our water scheme. (PO_014)	1	1	1	1	1	1	1	1	1	1	1	1
It is hard for me to think about this water scheme as mine. (PO_017)	0	0	0	0	0	0	0	0	0	0	0	0
Most of the people that live in this village feel as though they own the water scheme. (PO_020)	1	1	1	1	0	0	0	0	0	0	0	0

Note: *N* = 328. Based on the AISP table. Items 17 and 20 are excluded from the scale by selecting the remaining items that show unidimensionality at a threshold level of 0.30. Numbers represent which subscale the item belongs to; 0 indicates the item is unscalable at that homogeneity level. No multidimensional solution is apparent from this table: no groups of items identified as ‘leaving to form another scale’ at the same homogeneity threshold. Abbreviations: AISP = automatic item selection procedure; H = homogeneity.

**Table 7:** Monotonicity with default minimum size of a rest score group n = N/5.

	ItemH	#ac	#vi	#vi/#ac	maxvi	sum	sum/#ac	zmax	#zsig	crit
This is my water scheme. (PO_002)	0.53	21	2	0.10	0.05	0.09	0.00	0.80	0.00	15.00
I sense that this water scheme is our water scheme. (PO_005)	0.56	21	3	0.14	0.15	0.29	0.01	2.60	1.00	64.00
I feel a very high degree of personal ownership for this water scheme. (PO_008)	0.48	12	1	0.08	0.06	0.06	0.01	1.07	0.00	19.00
I sense that this is my water scheme. (PO_011)	0.57	12	0	0.00	0.00	0.00	0.00	0.00	0.00	0.00
This is our water scheme. (PO_014)	0.52	21	2	0.10	0.05	0.09	0.00	0.71	0.00	15.00

Note: *N* = 328. No significant violations were identified. Abbreviations: ItemH = Item homogeneity value; #ac = number of active pairs; #vi = number of violations; #vi/#ac = average number of violations per active pair; maxvi = largest violation of manifest monotonicity; sum = sum of violations of manifest monotonicity; sum/#ac = average violation per active pair; zmax = maximum z-value; #zsig = number of significant z-values; crit = crit value.

**Table 8:** Invariant item ordering (IIO) tests with default minimum size of a rest score group n = N/5.

	ItemH	#ac	#vi	#vi/#ac	maxvi	sum	sum/#ac	zmax	#zsig	crit
This is my water scheme. (PO_002)	0.53	11.00	1.00	0.09	0.19	0.19	0.02	0.65	0.00	42.00
I sense that this water scheme is our water scheme. (PO_005)	0.56	10.00	2.00	0.20	0.26	0.45	0.04	0.89	0.00	90.00
I feel a very high degree of personal ownership for this water scheme. (PO_008)	0.48	8.00	1.00	0.12	0.51	0.51	0.06	1.90	1.00	158.00
I sense that this is my water scheme. (PO_011)	0.57	9.00	2.00	0.22	0.51	0.57	0.06	1.90	1.00	161.00
This is our water scheme. (PO_014)	0.52	10.00	2.00	0.20	0.26	0.32	0.03	0.89	0.00	78.00

Note: *N* = 328. No significant violations were identified. Abbreviations: ItemH = Item homogeneity value; #ac = number of active pairs; #vi = number of violations; #vi/#ac = average number of violations per active pair; maxvi = largest violation of manifest monotonicity; sum = sum of violations of manifest monotonicity; sum/#ac = average violation per active pair; zmax = maximum z-value; #zsig = number of significant z-values; crit = crit value.

**Table 9:** Invariant item ordering (IIO) tests with default minimum size of a rest score group n = N/5 after deletion of most critical item (PO_008).

	ItemH	#ac	#vi	#vi/#ac	maxvi	sum	sum/#ac	zmax	#zsig	crit
This is my water scheme. (PO_002)	0.51	6.00	1.00	0.17	0.06	0.06	0.01	0.24	0.00	25.00
I sense that this water scheme is our water scheme. (PO_005)	0.60	6.00	3.00	0.50	0.19	0.43	0.07	0.86	0.00	138.00
I sense that this is my water scheme. (PO_011)	0.59	5.00	1.00	0.20	0.18	0.18	0.04	0.86	0.00	69.00
This is our water scheme. (PO_014)	0.57	5.00	1.00	0.20	0.19	0.19	0.04	0.75	0.00	72.00

Note: *N* = 328. No significant violations were identified. Abbreviations: ItemH = Item homogeneity value; #ac = number of active pairs; #vi = number of violations; #vi/#ac = average number of violations per active pair; maxvi = largest violation of manifest monotonicity; sum = sum of violations of manifest monotonicity; sum/#ac = average violation per active pair; zmax = maximum z-value; #zsig = number of significant z-values; crit = crit value.

**Table 10:** Reliability indicators for all items.

	*Cronbach’s α* *[Lower CI—Upper CI]*	*Cronbach’s α Raw. if Item Dropped*	*McDonald’s Ω* *[Lower CI—Upper CI]*	*Revelle’s Ω*	*GLB*	*Lambda*
**Psychological ownership for the water system at baseline**	0.83 [0.8–0.86]		0.83 [0.8–0.86]	0.89	0.89	0.83
This is my water scheme. (PO_002)		0.82				
I sense that this water scheme is our water scheme. (PO_005)		0.76				
I sense that this is my water scheme. (PO_011)		0.77				
This is our water scheme. (PO_014)		0.77				

Note: *N* = 328.; Abbreviations: CI = confidence interval; GLB = greatest lower bound.

**Table 11:** Descriptive statistics of original and shortened scales at measurement time point 1.

	*M*	*SD*	*f*	*f%*	Coding
**Psychological ownership for the water system at baseline (7 items. original)**	3.49	1.04			
**Psychological ownership for the water system at baseline (4 items)**	3.17	0.82			1–5
			18	5.49	1
			6	1.83	1.25
			8	2.44	1.5
			14	4.27	1.75
			16	4.88	2
			9	2.74	2.25
			14	4.27	2.5
			13	3.96	2.75
			14	4.27	3
			10	3.05	3.25
			16	4.88	3.5
			7	2.13	3.75
			148	45.12	4
			16	4.88	4.25
			10	3.05	4.5
			4	1.22	4.75
			5	1.52	5

Note: *N* = 328. *f* = absolute frequency. *f*% = relative frequency. *M* = Mean; *SD* = standard deviation.

## Data Availability

Data can be provided upon request.

## Supplementary Materials

The following supporting information can be downloaded at: https://www.mdpi.com/article/10.3390/ijerph191912775/s1, S1: Interview guideline grounded theory; S2: Interview guideline think aloud reasoning; S3: Questionnaire; S4: Fit indices of three dimensional psychological ownership scale.
