# Supplementary material for: Contextualized Measurement Scale Adaptation: A 4-Step Tutorial for Health Psychology Research
Source: Int J Environ Res Public Health. 2022 Oct 6;19(19):12775. doi: 10.3390/ijerph191912775 (PMC9566381; doi:10.3390/ijerph191912775)
Supplement: Supplementary file 1 [file ijerph-19-12775-s001.zip › ijerph-1886828-supplementary.pdf]

Supplementary materials to:
4-Step Protocol for Contextual Adaptation of Measurement Instruments

<u>Supplementary materials S1:</u>

Interview guideline grounded theory

Water concerns

- 3) What are the water related problems you are most concerned about?

In the following section the main goal is to have a conversation with the respondent. There are questions (indicated in bold) which have to be asked in this very exact way. And then there are a lot of sub-questions prepared to sum up, what the content of the response should include. The goal now is to let the respondent talk freely and provide us with the information that comes into his mind and at the same time to lead the talk so that we get what we want to learn.

Organization of water system

In this first part of the interview, we'd like to learn from you about the water supply in your village.

- 5) Can you tell me, how water supply is organized?
  - How is the water system during dry season? How is it during rainy season?
  - Who is responsible for collecting water?
  - How many times do you collect water a day at this source?
  - When do you collect the water?
  - What triggers you to go and collect water? What do you do most of the times directly before collecting water?

- 4) What is your first thought when you think of water collection?
  - Where is the closest community filter for you?
  - Why do/don't you collect water there?

o Who decided that you collect/don’t collect water there?

- 7) What is the procedure when you are going to collect water?

o How long does it take you to collect water?

o Who else uses this water source?

o Who takes care of this water source?

o Do you meet other people when collecting water?

o What are the benefits of using this water source?

o What are the disadvantages of using this water source?

- 8) How is this system organized? Who is responsible for which task?

o How much does it cost to collect water?

o Do you think this is a fair fare?

o How much do you pay for maintenance of this water source?

o Who else pays for maintenance of this water source?

o How much did you pay for the installation of this water source?

o Who installed this water source?

o How was the system installed? Can you tell me, how this came about?

o Who looks after the system?

o Who may use the water system? Who may not use it?

- How many people are using the filter in your community?
- What happened to the water before collecting?

- 9) How do you rate the organization of this system?
  - How easy is it to access and to use?
  - What could be better?
  - What is the most preferred water collection option for you? Why?
  - If you saw someone damaging or vandalizing the water system, what would you do?
  - How IMPORTANT would you say the water filter is to the LIVELIHOODS of you and your family?
  - How IMPORTANT would you say the water system is to the LIVELIHOODS of people in this village?
  - What else is important for the LIVELIHOODS of people in this village?

- 10) What was there before?
  - Were you ever visited by people from NGOs or the government who talked to you about water-related issues?
  - What kind of community or group activity regarding water-related issues have been going on in your village?
  - What were your fears and hopes before they started the project? What are your feelings now?
  - How did you get the filter?
  - Who was involved in the construction?
  - How useful is it to collect water at this source?
  - How easy is it to use?

- What are barriers for using this water source?
- What would make the use more convenient for you?
- What water source did you use before?
- What is better now? What was better before?

- 11a) What do you think of the water you collect?

- Do you believe this water source is safe?
- Has this source ever been tested for arsenic?
- By whom?
- Is this source contaminated with other things (e.g. bacteria)?
- Has this source ever been tested for other indicators of water quality?
- For which ones?
- Is this source contaminated with arsenic?
- Do you think you are at low or high risk to develop an illness when drinking water from this source?
- How high/low do you think is the risk?
- What is the risk?
- Do you think there is something harmful in the water collected from this source?
- What is it?
- What does it do that you perceive as harmful?
- How does it get into the water?
- How can you clean the water?

o Which of these substances does the water from this source contain?

o Is it necessary to purify the water form this substance?

o How can you purify it?

o Do you think there is something harmful in the food you cooked with this water?

o What is it?

o What does it do that you perceive as harmful?

o How does it get into the water?

o How can you clean the water?

o Which of these substances does the food cooked with water from this source contain?

o Is it necessary to purify the water form this substance?

o How can you purify it?

- 11b) How would a water-collection option best be organized for you?

Ownership towards this water system

- 122) Can you give me some examples of what you would say, that you own?

- 121) In your own words, can you tell me what you feel, when you think about these things?

o Can you imagine not actually owning something, but feeling like it’s yours?

o What is important to you to feel a certain level of security over a thing: investing the self, knowledge or being in control?

- What is the meaning or the importance of owning?
- What advantages has it, that it is your own?
- What disadvantages has it, that it is your own?
- What items belong to the members of your family together? What items belong to the members of your village together?
- Can you tell me why/how something just belongs to you and other things to your family or village? What is the difference?

- 13) You said you feel you are / are not one of the onwers of the community water filter. Can you explain what makes you feel that the community filter belongs / doesn't belong to you?

- Who decided or decides about the community filter? Can you influence this?
- Can you explain to me, how the drinking water system works? Can you explain me, how the filter works?
- How much did you or how much do you invest into the system?

14) And what are the consequences, when you say the community filter is your own?

- Let's think of the drinking water system in your village: Can you explain, who owns it? Who is responsible for ensuring the system works properly? Do you feel responsible?
- What are your responsibilities for the functioning of the community filter?

  E.g. paying money for maintenance and use, making repairs, perhaps also social responsibilities etc.
- What rights do you have when it comes to the community filter?

E.g. using it anytime you like, or fixed amount of water you can get etc.

o Who else is responsible for the community filter?

What are their tasks? How well are they doing them?

o Individual and collective ownership – what are your different associations with possession of yourself and shared possession? How do you distinguish these two levels?

Supplementary materials to:
4-Step Protocol for Contextual Adaptation of Measurement Instruments

Supplementary materials S2:

Interview guideline think aloud reasoning

I am now reading you a couple of statements and sentences on your water system, which you can please complete or tell us what goes through your head. Please think aloud – tell us everything that goes through your mind. It is very important to emphasize that you cannot say anything wrong. What you are thinking is what we are interested in.

- 151: This is my water system.
- 152: This is our community's water system.
- 153: It is hard for me to think about this water system as mine.
- 154: My family is one of the owners of the water system.
- 155. The water system is owned by all the people who live in this village
- 156. This is my filter
- 157. This is OUR FAMILY`s water filter
- 158. It is hard for me to think about this water filter as MINE.

- 159. MY favourite water collection option is…

- 1510. MY FAMILY's favourite water collection option is…

- 1511. OUR favourite water collection option is…

Supplementary materials to:
4-Step Protocol for Contextual Adaptation of Measurement Instruments

Supplementary materials S3:

Questionnaire

The questionnaire with all the items included in the survey is attached here.

English label text	**choices code**	choices English
Hello, my name is ………………………….. and I am a volonteer for Paridhi. In collaboration with Eawag, the Swiss Federal Institute of Aquatic Science and Technology, we are conducting a community based action on water consumption. If you don't mind, I would like to interview you about your water		

consumption preferences. We are also interviewing other households in your community as well as other communities in Bhagalpur District. It will take about 1 hour to complete the interview. Do you have the time for the interview? We are not interested in any particular answers, just in the answers that really represent your opinion. Your answers will be treated anonymously. We would like to know why people are doing what they are doing so that we can improve the

drinking water situation depending on this information in villages in Bhagalpur District. It helps us most if you answer as honest and properly as possible. Please help us in finding out how things really are!

Would you like to proceed with the interview?	1	yes
	0	no

Thank you for your interest, anyway! I wish you a good day.

Enter household-ID:

What is your name?

What is your name, other villagers call you?		
What is the name of the household's head?		
What is the main landmark of this house?		
How can we find you most easily?	1	call
	2	ask in the village for you
	3	see you at the house
After the rainy season, we will come and interview again, if you agree. To find you as easy as possible - please provide us with your phone number!		
To whom belongs this number?	1	the household of the respondent
	2	relatives, neighbours, friends
	3	no phone number
How old are you?		
Observe: Sex?	1	female
	2	male

	0	other
What is the religion of the household?	1	Hinduism
	2	Buddhism
	3	Islam
	4	Other
If other, please specify:		
To which caste do you belong to?	1	Upper Caste
	2	General Caste
	3	OBC
	4	Scheduled Tribe
	5	Scheduled Caste
What is your highest educational degree?	7	Profession or honours
	6	Graduate or postgraduate
	5	Intermediate or post high school diploma
	4	High school certificate
	3	Middle school certificate
	2	Primary school certificate
	1	Literate
	0	Illiterate
What is the head of household's highest	7	Profession or honours

educational degree?		
	6	Graduate or postgraduate
	5	Intermediate or post high school diploma
	4	High school certificate
	3	Middle school certificate
	2	Primary school certificate
	1	Literate
	0	Illiterate
Are you able to read?	1	yes
	0	no
Are you able to write?	1	yes
	0	no
What is your occupation?	10	Profession or honours
	6	Semi-Profession
	5	Clerical, Shop-owner
	4	Skilled worker
	3	Semi-skilled worker
	2	Unskilled worker / Farmer
	1	Unemployed / Housekeeper
What is the head of household's occupation?	10	Profession or honours
	6	Semi-Profession
	5	Clerical, Shop-owner
	4	Skilled worker
	3	Semi-skilled worker

	2	Unskilled worker / Farmer
	1	Unemployed / Housekeeper
Is this your own house?	1	yes
	0	no
If no: Who owns this house? What is your relation to the head of the household?	1	wife
	2	husband
	3	daughter
	4	mother
	5	sister
	6	son
	7	father
	8	brother
	9	grandson
	10	granddaughter
	11	I am the head of the household
What is the monthly income of the family?	12	more than 41430 INR
	10	20715-41429 INR
	6	15536-20714 INR
	4	10357-15535 INR
	3	6214-10356 INR
	2	2092-6213 INR
	1	less than 2091 INR

How much land does your household own (incl. homestead land}?

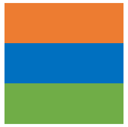

Think about the home or the cow or the bike or the mobile that you own and the experiences and feelings associated with the statement 'this is my home!', 'this is my cow!'. 'this is my bike!', 'this is my mobile!'. The following questions deal with the 'sense of ownership' that you feel for the water scheme that you have in this community. Indicate the

degree to which you personally agree or disagree with the following statements:		
This is my water scheme.	10	I agree.
	20	I disagree.
	3	I neither agree nor disagree.
How much do you agree?	4	I strongly agree.
	5	I agree a little.
How much do you disagree?	2	I disagree a little.
	1	I strongly disagree.
I sense that this water scheme is our water scheme.	10	I agree.
	20	I disagree.
	3	I neither agree nor disagree.
How much do you agree?	4	I strongly agree.
	5	I agree a little.
How much do you disagree?	2	I disagree a little.
	1	I strongly disagree.
I feel a very high degree of	10	I agree.

personal ownership for this water scheme.		
	20	I disagree.
	3	I neither agree nor disagree.
How much do you agree?	4	I strongly agree.
	5	I agree a little.
How much do you disagree?	2	I disagree a little.
	1	I strongly disagree.
I sense that this is my water scheme.	10	I agree.
	20	I disagree.
	3	I neither agree nor disagree.
How much do you agree?	4	I strongly agree.
	5	I agree a little.
How much do you disagree?	2	I disagree a little.
	1	I strongly disagree.
This is our water scheme.	10	I agree.
	20	I disagree.
	3	I neither agree nor disagree.
How much do you agree?	4	I strongly agree.
	5	I agree a little.

How much do you disagree?	2	I disagree a little.
	1	I strongly disagree.
It is hard for me to think about this water scheme as mine.	10	I agree.
	20	I disagree.
	3	I neither agree nor disagree.
How much do you agree?	4	I strongly agree.
	5	I agree a little.
How much do you disagree?	2	I disagree a little.
	1	I strongly disagree.
Most of the people that live in this village feel as though they own the water scheme.		
	20	I disagree.
	3	I neither agree nor disagree.
How much do you agree?	4	I strongly agree.
	5	I agree a little.
How much do you disagree?	2	I disagree a little.
	1	I strongly disagree.

I feel like I own the water scheme.	10	I agree.
	20	I disagree.
	3	I neither agree nor disagree.
How much do you agree?	4	I strongly agree.
	5	I agree a little.
How much do you disagree?	2	I disagree a little.
	1	I strongly disagree.
I have rights on the water scheme, that no one else has.	10	I agree.
	20	I disagree.
	3	I neither agree nor disagree.
How much do you agree?	4	I strongly agree.
	5	I agree a little.
How much do you disagree?	2	I disagree a little.
	1	I strongly disagree.
If something happens to this water scheme, others think that I am responsible for the water scheme.		
	20	I disagree.

	3	I neither agree nor disagree.
How much do you agree?	4	I strongly agree.
	5	I agree a little.
How much do you disagree?	2	I disagree a little.
	1	I strongly disagree.
I feel I have autonomy over the water scheme.	10	I agree.
	20	I disagree.
	3	I neither agree nor disagree.
How much do you agree?	4	I strongly agree.
	5	I agree a little.
How much do you disagree?	2	I disagree a little.
	1	I strongly disagree.
The feelings I have towards the water scheme are unique and cannot be matched by the feelings somebody else has towards the water scheme.		
	20	I disagree.
	3	I neither agree nor disagree.

How much do you agree?	4	I strongly agree.
	5	I agree a little.
How much do you disagree?	2	I disagree a little.
	1	I strongly disagree.
If others use the water scheme they have to follow my ideas of how to use.		
	20	I disagree.
	3	I neither agree nor disagree.
How much do you agree?	4	I strongly agree.
	5	I agree a little.
How much do you disagree?	2	I disagree a little.
	1	I strongly disagree.
I feel the water scheme belongs to all community members in my village.	10	I agree.
	20	I disagree.
	3	I neither agree nor disagree.
How much do you agree?	4	I strongly agree.
	5	I agree a little.
How much do you disagree?	2	I disagree a little.

	1	I strongly disagree.
It bothers me, when other people collect their water from the water system.		
	20	I disagree.
	3	I neither agree nor disagree.
How much do you agree?	4	I strongly agree.
	5	I agree a little.
How much do you disagree?	2	I disagree a little.
	1	I strongly disagree.
Other community members think that this is my water system.	10	I agree.
	20	I disagree.
	3	I neither agree nor disagree.
How much do you agree?	4	I strongly agree.
	5	I agree a little.
How much do you disagree?	2	I disagree a little.
	1	I strongly disagree.
I like the water scheme.	10	I agree.
	20	I disagree.
	3	I neither agree nor disagree.

How much do you agree?	4	I strongly agree.
	5	I agree a little.
How much do you disagree?	2	I disagree a little.
	1	I strongly disagree.
The water scheme is very important to me.	10	I agree.
	20	I disagree.
	3	I neither agree nor disagree.
How much do you agree?	4	I strongly agree.
	5	I agree a little.
How much do you disagree?	2	I disagree a little.
	1	I strongly disagree.
I feel that I need to own this water scheme.	10	I agree.
	20	I disagree.
	3	I neither agree nor disagree.
How much do you agree?	4	I strongly agree.
	5	I agree a little.
How much do you disagree?	2	I disagree a little.
	1	I strongly disagree.
I am passionate about the water scheme.	10	I agree.

	20	I disagree.
	3	I neither agree nor disagree.
How much do you agree?	4	I strongly agree.
	5	I agree a little.
How much do you disagree?	2	I disagree a little.
	1	I strongly disagree.
I feel that the water scheme belongs to me.	10	I agree.
	20	I disagree.
	3	I neither agree nor disagree.
How much do you agree?	4	I strongly agree.
	5	I agree a little.
How much do you disagree?	2	I disagree a little.
	1	I strongly disagree.
If the water scheme is broken, I feel personally sad.	10	I agree.
	20	I disagree.
	3	I neither agree nor disagree.
How much do you agree?	4	I strongly agree.
	5	I agree a little.
How much do you disagree?	2	I disagree a little.

	1	I strongly disagree.
Thinking of the water scheme gives me a positivie feeling.	10	I agree.
	20	I disagree.
	3	I neither agree nor disagree.
How much do you agree?	4	I strongly agree.
	5	I agree a little.
How much do you disagree?	2	I disagree a little.
	1	I strongly disagree.
The water scheme has no special meaning for me.	10	I agree.
	20	I disagree.
	3	I neither agree nor disagree.
How much do you agree?	4	I strongly agree.
	5	I agree a little.
How much do you disagree?	2	I disagree a little.
	1	I strongly disagree.
I feel the need to defend the water scheme when it is criticized by others.		
	20	I disagree.

	3	I neither agree nor disagree.
How much do you agree?	4	I strongly agree.
	5	I agree a little.
How much do you disagree?	2	I disagree a little.
	1	I strongly disagree.
I am proud to tell others of the water scheme.	10	I agree.
	20	I disagree.
	3	I neither agree nor disagree.
How much do you agree?	4	I strongly agree.
	5	I agree a little.
How much do you disagree?	2	I disagree a little.
	1	I strongly disagree.
It is important to me that others think highly of the water scheme.	10	I agree.
	20	I disagree.
	3	I neither agree nor disagree.
How much do you agree?	4	I strongly agree.
	5	I agree a little.
How much do you disagree?	2	I disagree a little.
	1	I strongly disagree.

It is important to me to support the water scheme.	10	I agree.
	20	I disagree.
	3	I neither agree nor disagree.
How much do you agree?	4	I strongly agree.
	5	I agree a little.
How much do you disagree?	2	I disagree a little.
	1	I strongly disagree.
My wellbeing is linked to the functionality of the water scheme.	10	I agree.
	20	I disagree.
	3	I neither agree nor disagree.
How much do you agree?	4	I strongly agree.
	5	I agree a little.
How much do you disagree?	2	I disagree a little.
	1	I strongly disagree.
I consider problems at the water scheme as my own problems.	10	I agree.
	20	I disagree.
	3	I neither agree nor disagree.

How much do you agree?	4	I strongly agree.
	5	I agree a little.
How much do you disagree?	2	I disagree a little.
	1	I strongly disagree.
This water scheme or a piece of this water scheme is really mine.	10	I agree.
	20	I disagree.
	3	I neither agree nor disagree.
How much do you agree?	4	I strongly agree.
	5	I agree a little.
How much do you disagree?	2	I disagree a little.
	1	I strongly disagree.
	20	I disagree.
	3	I neither agree nor disagree.
How much do you agree?	4	I strongly agree.
	5	I agree a little.
How much do you disagree?	2	I disagree a little.
	1	I strongly disagree.
If I were to describe myself, this water scheme would		

likely be something I would mention		
	20	I disagree.
	3	I neither agree nor disagree.
How much do you agree?	4	I strongly agree.
	5	I agree a little.
How much do you disagree?	2	I disagree a little.
	1	I strongly disagree.
I feel that I love the water system	10	I agree.
	20	I disagree.
	3	I neither agree nor disagree.
How much do you agree?	4	I strongly agree.
	5	I agree a little.
How much do you disagree?	2	I disagree a little.
	1	I strongly disagree.
I feel great affection for the water system	10	I agree.
	20	I disagree.
	3	I neither agree nor disagree.
How much do you agree?	4	I strongly agree.
	5	I agree a little.

How much do you disagree?	2	I disagree a little.
	1	I strongly disagree.
I feel like it is in my hand who can use the water system and who can not		
	20	I disagree.
	3	I neither agree nor disagree.
How much do you agree?	4	I strongly agree.
	5	I agree a little.
How much do you disagree?	2	I disagree a little.
	1	I strongly disagree.
I feel like it is in my hand how often I may use the water system		
	20	I disagree.
	3	I neither agree nor disagree.
How much do you agree?	4	I strongly agree.
	5	I agree a little.
How much do you disagree?	2	I disagree a little.
	1	I strongly disagree.
I would challenge anyone in my community if I		

thought something with the water system was done wrong.		
	20	I disagree.
	3	I neither agree nor disagree.
How much do you agree?	4	I strongly agree.
	5	I agree a little.
How much do you disagree?	2	I disagree a little.
	1	I strongly disagree.
I know best, when something has to be decided over the water scheme.	10	I agree.
	20	I disagree.
	3	I neither agree nor disagree.
How much do you agree?	4	I strongly agree.
	5	I agree a little.
How much do you disagree?	2	I disagree a little.
	1	I strongly disagree.
I am the head of the water scheme.	10	I agree.
	20	I disagree.
	3	I neither agree nor disagree.

How much do you agree?	4	I strongly agree.
	5	I agree a little.
How much do you disagree?	2	I disagree a little.
	1	I strongly disagree.
I am one of the leaders over the water scheme.	10	I agree.
	20	I disagree.
	3	I neither agree nor disagree.
How much do you agree?	4	I strongly agree.
	5	I agree a little.
How much do you disagree?	2	I disagree a little.
	1	I strongly disagree.
I know how this water scheme is organised.	10	I agree.
	20	I disagree.
	3	I neither agree nor disagree.
How much do you agree?	4	I strongly agree.
	5	I agree a little.
How much do you disagree?	2	I disagree a little.
	1	I strongly disagree.
I know how this water scheme works.	10	I agree.

	20	I disagree.
	3	I neither agree nor disagree.
How much do you agree?	4	I strongly agree.
	5	I agree a little.
How much do you disagree?	2	I disagree a little.
	1	I strongly disagree.
I know who is responsible for the water scheme if there are any troubles.	10	I agree.
	20	I disagree.
	3	I neither agree nor disagree.
How much do you agree?	4	I strongly agree.
	5	I agree a little.
How much do you disagree?	2	I disagree a little.
	1	I strongly disagree.
I am familiar with the purpose of this water scheme.	10	I agree.
	20	I disagree.
	3	I neither agree nor disagree.
How much do you agree?	4	I strongly agree.
	5	I agree a little.

How much do you disagree?	2	I disagree a little.
	1	I strongly disagree.
I take every opportunity to oversee how things are operated in this water scheme.		
	20	I disagree.
	3	I neither agree nor disagree.
How much do you agree?	4	I strongly agree.
	5	I agree a little.
How much do you disagree?	2	I disagree a little.
	1	I strongly disagree.
I have control over the water scheme.	10	I agree.
	20	I disagree.
	3	I neither agree nor disagree.
How much do you agree?	4	I strongly agree.
	5	I agree a little.
How much do you disagree?	2	I disagree a little.
	1	I strongly disagree.
I organise the water scheme to better align it	10	I agree.

with my water-collection practice.		
	20	I disagree.
	3	I neither agree nor disagree.
How much do you agree?	4	I strongly agree.
	5	I agree a little.
How much do you disagree?	2	I disagree a little.
	1	I strongly disagree.
I was not at all involved in the planning of the water scheme.	10	I agree.
	20	I disagree.
	3	I neither agree nor disagree.
How much do you agree?	4	I strongly agree.
	5	I agree a little.
How much do you disagree?	2	I disagree a little.
	1	I strongly disagree.
I cannot influence what happens with the water scheme.	10	I agree.
	20	I disagree.
	3	I neither agree nor disagree.
How much do you agree?	4	I strongly agree.

	5	I agree a little.
How much do you disagree?	2	I disagree a little.
	1	I strongly disagree.
I strongly influenced the decision, where the water scheme was going to be built.	10	I agree.
	20	I disagree.
	3	I neither agree nor disagree.
How much do you agree?	4	I strongly agree.
	5	I agree a little.
How much do you disagree?	2	I disagree a little.
	1	I strongly disagree.
My family invested money or donated land in the water scheme.	10	I agree.
	20	I disagree.
	3	I neither agree nor disagree.
How much do you agree?	4	I strongly agree.
	5	I agree a little.
How much do you disagree?	2	I disagree a little.
	1	I strongly disagree.

My family invested labour in the water scheme.	10	I agree.
	20	I disagree.
	3	I neither agree nor disagree.
How much do you agree?	4	I strongly agree.
	5	I agree a little.
How much do you disagree?	2	I disagree a little.
	1	I strongly disagree.
Without my contribution, the water scheme would not exist.	10	I agree.
	20	I disagree.
	3	I neither agree nor disagree.
How much do you agree?	4	I strongly agree.
	5	I agree a little.
How much do you disagree?	2	I disagree a little.
	1	I strongly disagree.
I accept the water scheme as my water scheme.	10	I agree.
	20	I disagree.
	3	I neither agree nor disagree.
How much do you agree?	4	I strongly agree.

	5	I agree a little.
How much do you disagree?	2	I disagree a little.
	1	I strongly disagree.
They told me the water scheme was mine.	10	I agree.
	20	I disagree.
	3	I neither agree nor disagree.
How much do you agree?	4	I strongly agree.
	5	I agree a little.
How much do you disagree?	2	I disagree a little.
	1	I strongly disagree.
They told me I was responsible for the water scheme.	10	I agree.
	20	I disagree.
	3	I neither agree nor disagree.
How much do you agree?	4	I strongly agree.
	5	I agree a little.
How much do you disagree?	2	I disagree a little.
	1	I strongly disagree.
The water collected at the water scheme, is of good quality		

and drinkable and usable for cooking purpose.		
	20	I disagree.
	3	I neither agree nor disagree.
How much do you agree?	4	I strongly agree.
	5	I agree a little.
How much do you disagree?	2	I disagree a little.
	1	I strongly disagree.
The community water scheme is very important for me.	10	I agree.
	20	I disagree.
	3	I neither agree nor disagree.
How much do you agree?	4	I strongly agree.
	5	I agree a little.
How much do you disagree?	2	I disagree a little.
	1	I strongly disagree.
When the water scheme is not in use, I don't care.	10	I agree.
	20	I disagree.
	3	I neither agree nor disagree.
How much do you agree?	4	I strongly agree.

	5	I agree a little.
How much do you disagree?	2	I disagree a little.
	1	I strongly disagree.
Every other water source is second to the water scheme.	10	I agree.
	20	I disagree.
	3	I neither agree nor disagree.
How much do you agree?	4	I strongly agree.
	5	I agree a little.
How much do you disagree?	2	I disagree a little.
	1	I strongly disagree.
I benefit from the water scheme financially.	10	I agree.
	20	I disagree.
	3	I neither agree nor disagree.
How much do you agree?	4	I strongly agree.
	5	I agree a little.
How much do you disagree?	2	I disagree a little.
	1	I strongly disagree.
The water scheme is good for me and my family.	10	I agree.

	20	I disagree.
	3	I neither agree nor disagree.
How much do you agree?	4	I strongly agree.
	5	I agree a little.
How much do you disagree?	2	I disagree a little.
	1	I strongly disagree.
News about the water scheme are big news for me.	10	I agree.
	20	I disagree.
	3	I neither agree nor disagree.
How much do you agree?	4	I strongly agree.
	5	I agree a little.
How much do you disagree?	2	I disagree a little.
	1	I strongly disagree.
I perceive myself as very effective, if I would like to change something at the water scheme.	10	I agree.
	20	I disagree.
	3	I neither agree nor disagree.
How much do you agree?	4	I strongly agree.
	5	I agree a little.

How much do you disagree?	2	I disagree a little.
	1	I strongly disagree.
I can do whatever I want for the water scheme, I will not achieve anything.		
	20	I disagree.
	3	I neither agree nor disagree.
How much do you agree?	4	I strongly agree.
	5	I agree a little.
How much do you disagree?	2	I disagree a little.
	1	I strongly disagree.
When I want to collect water at the water scheme, I use it anytime at my convenience.		
	20	I disagree.
	3	I neither agree nor disagree.
How much do you agree?	4	I strongly agree.
	5	I agree a little.
How much do you disagree?	2	I disagree a little.
	1	I strongly disagree.

The water scheme makes me feeling strong and influencial.	10	I agree.
	20	I disagree.
	3	I neither agree nor disagree.
How much do you agree?	4	I strongly agree.
	5	I agree a little.
How much do you disagree?	2	I disagree a little.
	1	I strongly disagree.
If I wanted, I could ignore other peoples claims and have it as I like.		
	20	I disagree.
	3	I neither agree nor disagree.
How much do you agree?	4	I strongly agree.
	5	I agree a little.
How much do you disagree?	2	I disagree a little.
	1	I strongly disagree.
I feel I need to protect the water scheme.	10	I agree.
	20	I disagree.
	3	I neither agree nor disagree.

How much do you agree?	4	I strongly agree.
	5	I agree a little.
How much do you disagree?	2	I disagree a little.
	1	I strongly disagree.
I feel that other villagers should not use the water scheme.	10	I agree.
	20	I disagree.
	3	I neither agree nor disagree.
How much do you agree?	4	I strongly agree.
	5	I agree a little.
How much do you disagree?	2	I disagree a little.
	1	I strongly disagree.
Other people mustn't handle the water scheme.	10	I agree.
	20	I disagree.
	3	I neither agree nor disagree.
How much do you agree?	4	I strongly agree.
	5	I agree a little.
How much do you disagree?	2	I disagree a little.
	1	I strongly disagree.

I feel that we in the village all have the same rights on the water scheme.	10	I agree.
	20	I disagree.
	3	I neither agree nor disagree.
How much do you agree?	4	I strongly agree.
	5	I agree a little.
How much do you disagree?	2	I disagree a little.
	1	I strongly disagree.
Everybody can use the water scheme equally.	10	I agree.
	20	I disagree.
	3	I neither agree nor disagree.
How much do you agree?	4	I strongly agree.
	5	I agree a little.
How much do you disagree?	2	I disagree a little.
	1	I strongly disagree.
We are all obliged to contribute equally to repairing and maintenance of the water scheme.		

	20	I disagree.
	3	I neither agree nor disagree.
How much do you agree?	4	I strongly agree.
	5	I agree a little.
How much do you disagree?	2	I disagree a little.
	1	I strongly disagree.
I really care about the fate of the water scheme.	10	I agree.
	20	I disagree.
	3	I neither agree nor disagree.
How much do you agree?	4	I strongly agree.
	5	I agree a little.
How much do you disagree?	2	I disagree a little.
	1	I strongly disagree.
When the water scheme is damaged, I try my best to have it repaired.		
	20	I disagree.
	3	I neither agree nor disagree.
How much do you agree?	4	I strongly agree.
	5	I agree a little.

How much do you disagree?	2	I disagree a little.
	1	I strongly disagree.
I often motivate other people to take care of the water scheme.	10	I agree.
	20	I disagree.
	3	I neither agree nor disagree.
How much do you agree?	4	I strongly agree.
	5	I agree a little.
How much do you disagree?	2	I disagree a little.
	1	I strongly disagree.
I am sure, the water scheme will be broken in the future.	10	I agree.
	20	I disagree.
	3	I neither agree nor disagree.
How much do you agree?	4	I strongly agree.
	5	I agree a little.
How much do you disagree?	2	I disagree a little.
	1	I strongly disagree.
I think the water scheme provides me with safe	10	I agree.

water in the next 4 weeks.		
	20	I disagree.
	3	I neither agree nor disagree.
How much do you agree?	4	I strongly agree.
	5	I agree a little.
How much do you disagree?	2	I disagree a little.
	1	I strongly disagree.
I feel responsible for the water scheme.	10	I agree.
	20	I disagree.
	3	I neither agree nor disagree.
How much do you agree?	4	I strongly agree.
	5	I agree a little.
How much do you disagree?	2	I disagree a little.
	1	I strongly disagree.
Do you feel responsible for the repairing of the water system in case of interruption?	10	I agree.
	20	I disagree.
	3	I neither agree nor disagree.

How much do you agree?	4	I strongly agree.
	5	I agree a little.
How much do you disagree?	2	I disagree a little.
	1	I strongly disagree.
I feel the duty to personally take care of the water system.		
	20	I disagree.
	3	I neither agree nor disagree.
How much do you agree?	4	I strongly agree.
	5	I agree a little.
How much do you disagree?	2	I disagree a little.
	1	I strongly disagree.
I feel the same, it doesn’t matter if I do maintain the water scheme or not.		
	20	I disagree.
	3	I neither agree nor disagree.
How much do you agree?	4	I strongly agree.
	5	I agree a little.
How much do you disagree?	2	I disagree a little.
	1	I strongly disagree.

There are valuable things in the water scheme to look after.	10	I agree.
	20	I disagree.
	3	I neither agree nor disagree.
How much do you agree?	4	I strongly agree.
	5	I agree a little.
How much do you disagree?	2	I disagree a little.
	1	I strongly disagree.
I am having rights on the water scheme.	10	I agree.
	20	I disagree.
	3	I neither agree nor disagree.
How much do you agree?	4	I strongly agree.
	5	I agree a little.
How much do you disagree?	2	I disagree a little.
	1	I strongly disagree.
I have to follow the instructions of the people that have rights on the water scheme.	10	I agree.
	20	I disagree.
	3	I neither agree nor disagree.

How much do you agree?

4 I strongly agree.

5 I agree a little.

How much do you disagree?

2 I disagree a little.

1 I strongly disagree.

Think about the temple, public schools, a public hospital, or some other item that you own or co-own with the community, and the experiences and feelings associated with the statement ‘THIS IS OURS!’ The following questions deal with the ‘feelings of ownership’ that you and your community members feel for

the water scheme in the village.		
We, my community members and I, collectively agree that this is OUR water scheme.		
	20	I disagree.
	3	I neither agree nor disagree.
How much do you agree?	4	I strongly agree.
	5	I agree a little.
How much do you disagree?	2	I disagree a little.
	1	I strongly disagree.
We, my community members and I, collectively feel that this water scheme belongs to US together.		
	20	I disagree.
	3	I neither agree nor disagree.
How much do you agree?	4	I strongly agree.

	5	I agree a little.
How much do you disagree?	2	I disagree a little.
	1	I strongly disagree.
We, my community members and I, feel a very high degree of collective (community} ownership for this water scheme.		
	20	I disagree.
	3	I neither agree nor disagree.
How much do you agree?	4	I strongly agree.
	5	I agree a little.
How much do you disagree?	2	I disagree a little.
	1	I strongly disagree.
All of the members of the community feel as though we own this water scheme collectively.		
	20	I disagree.
	3	I neither agree nor disagree.

How much do you agree?	4	I strongly agree.
	5	I agree a little.
How much do you disagree?	2	I disagree a little.
	1	I strongly disagree.
We as a community exercise influence over what is going on with the water scheme.		
	20	I disagree.
	3	I neither agree nor disagree.
How much do you agree?	4	I strongly agree.
	5	I agree a little.
How much do you disagree?	2	I disagree a little.
	1	I strongly disagree.
We as a community exercise control over what is going on with the water scheme.		
	20	I disagree.
	3	I neither agree nor disagree.
How much do you agree?	4	I strongly agree.
	5	I agree a little.

How much do you disagree?	2	I disagree a little.
	1	I strongly disagree.
We as a community were involved in the planning of the water scheme.	10	I agree.
	20	I disagree.
	3	I neither agree nor disagree.
How much do you agree?	4	I strongly agree.
	5	I agree a little.
How much do you disagree?	2	I disagree a little.
	1	I strongly disagree.
We as a community decided where the water scheme was going to be built.	10	I agree.
	20	I disagree.
	3	I neither agree nor disagree.
How much do you agree?	4	I strongly agree.
	5	I agree a little.
How much do you disagree?	2	I disagree a little.
	1	I strongly disagree.

Without the contribution of my community members and myself, the water scheme would not exist.	10	I agree.
	20	I disagree.
	3	I neither agree nor disagree.
How much do you agree?	4	I strongly agree.
	5	I agree a little.
How much do you disagree?	2	I disagree a little.
	1	I strongly disagree.
All community members and I, we invested money or land in the water scheme.	10	I agree.
	20	I disagree.
	3	I neither agree nor disagree.
How much do you agree?	4	I strongly agree.
	5	I agree a little.
How much do you disagree?	2	I disagree a little.
	1	I strongly disagree.
All community members and I,	10	I agree.

we invested labour in the water scheme.		
	20	I disagree.
	3	I neither agree nor disagree.
How much do you agree?	4	I strongly agree.
	5	I agree a little.
How much do you disagree?	2	I disagree a little.
	1	I strongly disagree.
All community members and I, we feel that we know the water system a lot.		
	20	I disagree.
	3	I neither agree nor disagree.
How much do you agree?	4	I strongly agree.
	5	I agree a little.
How much do you disagree?	2	I disagree a little.
	1	I strongly disagree.
All community members and I, we are informed about what is going on with the water scheme.		
	20	I disagree.

	3	I neither agree nor disagree.
How much do you agree?	4	I strongly agree.
	5	I agree a little.
How much do you disagree?	2	I disagree a little.
	1	I strongly disagree.
All community members and I, we know how this water scheme works.	10	I agree.
	20	I disagree.
	3	I neither agree nor disagree.
How much do you agree?	4	I strongly agree.
	5	I agree a little.
How much do you disagree?	2	I disagree a little.
	1	I strongly disagree.
In this community it happens a lot, that people defer responsibilities to other community members.		
	20	I disagree.
	3	I neither agree nor disagree.
How much do you agree?	4	I strongly agree.
	5	I agree a little.

How much do you disagree?	2	I disagree a little.
	1	I strongly disagree.
In this community it happens a lot, that someone does not do his or her share of the work.		
	20	I disagree.
	3	I neither agree nor disagree.
How much do you agree?	4	I strongly agree.
	5	I agree a little.
How much do you disagree?	2	I disagree a little.
	1	I strongly disagree.
In this community it happens a lot, that someone puts forth less effort than other community members.		
	20	I disagree.
	3	I neither agree nor disagree.
How much do you agree?	4	I strongly agree.
	5	I agree a little.
How much do you disagree?	2	I disagree a little.

	1	I strongly disagree.
No difficulty related the repairing of the water scheme is too tough for our community.		
	20	I disagree.
	3	I neither agree nor disagree.
How much do you agree?	4	I strongly agree.
	5	I agree a little.
How much do you disagree?	2	I disagree a little.
	1	I strongly disagree.
Our community has confidence in itself.	10	I agree.
	20	I disagree.
	3	I neither agree nor disagree.
How much do you agree?	4	I strongly agree.
	5	I agree a little.
How much do you disagree?	2	I disagree a little.
	1	I strongly disagree.
Our community expects to be known in other communities as highly caretaking		

of the water scheme.		
	20	I disagree.
	3	I neither agree nor disagree.
How much do you agree?	4	I strongly agree.
	5	I agree a little.
How much do you disagree?	2	I disagree a little.
	1	I strongly disagree.
The community's contribution to the water scheme is very valuable.	10	I agree.
	20	I disagree.
	3	I neither agree nor disagree.
How much do you agree?	4	I strongly agree.
	5	I agree a little.
How much do you disagree?	2	I disagree a little.
	1	I strongly disagree.
Our community delivers it important changes on the water scheme.	10	I agree.
	20	I disagree.
	3	I neither agree nor disagree.

How much do you agree?	4	I strongly agree.
	5	I agree a little.
How much do you disagree?	2	I disagree a little.
	1	I strongly disagree.
Our commuinity works on important problems related to the water scheme.	10	I agree.
	20	I disagree.
	3	I neither agree nor disagree.
How much do you agree?	4	I strongly agree.
	5	I agree a little.
How much do you disagree?	2	I disagree a little.
	1	I strongly disagree.

We have now halfway completed the interview. How do you feel? In the next parts of the questionnaire, we

are interested to learn from you about your water collection behaviour. This is, because we want to learn about the use and its reasons of water infrastructure like handpumps and community based water infrastructure. You help us most, with your answers to find out how people in Bhagalpur really collect water!		
Are you caretaker, user or non-user of the community water scheme?	1	Caretaker
	2	User
	3	Non-User
Why are you the caretaker?		
What is your main source for daily drinking and	1	private handpump

cooking water collection?		
	2	shared handpump
	3	filtration unit
	4	private borewell or private arrow filter
	5	public taps of community based piped water
	6	bottled water
	7	open well
	8	household tap of community based piped water scheme
How many times did you collect water from this main source per day for drinking and cooking purpose during the last 4 weeks?		
What is the volume of the bucket you collect the water with (INTERVIEWER: please verify the answer or measure the volume}?		
What is the main alternative source for daily drinking and cooking water collection		

that you used during the last 4 weeks?		
	2	shared handpump
	3	filtration unit
	4	private borewell or private arrow filter
	5	public taps of piped water
	6	bottled water
	7	open well
	8	household tap of community based piped water scheme
How many times did you collect water at this alternative source per day for drinking and cooking purpose during the last 4 weeks?		
What is the volume of the bucket you collect the water with (please check plausibility of the answer or measure the volume}?		
Multiple! Where do you collect drinking and		

cooking water during the monsoon (rainy season}?		
	2	shared handpump
	3	filtration unit
	4	private borewell or private arrow filter
	5	public taps of piped water
	6	bottled water
	7	open well
	8	household tap of community based piped water scheme
Do people of different castes collect drinking and cooking water at different sources?	0	no, we all collect water at the same source
	1	yes, some castes exclusively uses the water scheme, the other caste has to use other sources (e.g. open wells, handpumps, ...}
	2	yes, different castes have it's own water scheme
	3	yes, there are separate collecting points depending on which caste one belongs to
Has your handpump been tested on water-quality?	1	yes
	0	no
	999	I don't know
If yes, on what components?	1	fluoride
	2	iron

	3	bacterial contamination
	4	arsenic
Is it contaminated with arsenic?	1	yes
	0	no
	999	I don't know
How sure are you, that it is contaminated with arsenic?	5	extremely sure
	4	very sure
	3	I don't know
	2	a little sure
	1	not at all sure
How sure are you, that it is not contaminated with arsenic?	5	extremely sure
	4	very sure
	3	I don't know
	2	a little sure
	1	not at all sure
Are you willing or unwilling to help the caretaker to look after the community water scheme?		
	10	unwilling
	3	neither willing nor unwilling

How willing are you?	5	very much willing
	4	a little willing
How unwilling are you?	2	a little unwilling
	1	very much unwilling
Multiple! Which of the following tasks would you be ready to support the caretaker?		
	2	maintenance
	3	running
	4	repairing when broken
	5	changing the filtermedia
	6	testing the waterquality
Generally, do you think the chance that you get skin related diseases (such as changes in colour and hard patches on the palms & soles of the feet} if you drink water from		

the handpump is high or low?		
	10	High
	3	Neither low nor high.
how high do you think it is?	4	Somewhat high.
	5	Very high.
How low do you think it is?	2	Somewhat low.
	1	Very low.
Do you think the risk is high or low to get cancer when you drink water from the community water scheme only?		
	10	High
	3	Neither low nor high.
how high do you think it is?	4	Somewhat high.
	5	Very high.
How low do you think it is?	2	Somewhat low.
	1	Very low.
How good or bad is it for you and your health to drink water from		

the community water scheme?		
	10	Good
	3	Neither good nor bad
How good is it?	4	Somewhat good
	5	Very good
How bad is it?	2	Somewhat bad
	1	Very bad
How often do you think you need to drink water from the community water scheme in order to not experience any illness?		
	2	Most of the times
	3	Sometimes
	4	Few
	5	Never
Imagine you have skin related diseases (such as changes in colour and hard patches on the palms & soles of the feet}, how severe would be the		

impact on your life?	
	2 Not.<br>3 A little.<br>4 Strongly.<br>5 Very.
READ! Arsenic is a naturally existing toxic metal. It is especially present at high levels in the groundwater of gangetic plane (several States in India as well as Bangladesh}. Introduction: The questions and responses will be read to the study participant. The interviewer should circle the number corresponding to the subject's answer. Tell other individuals present during the quiz to not	

assist the respondent. Remind respondents that it is okay if they don't know an answer. However do not suggest that a respondent answer "I don't know" to any particular study question. Can you please tell me for each of the following whether it is a major effect of peoples exposure towards arsenic? Black dots on the skin is an effect of arsenic exposure.	1	yes
	0	no
	999	I don't know
Stomach problems and problems with digestion are effects of arsenic exposure.	1	yes

	0	no
	999	I don't know
Changes in thickness and surface of the skin on hands, feet and the back are effects of arsenic exposure.		
	0	no
	999	I don't know
Diarrhea is an effect of arsenic exposure.	1	yes
	0	no
	999	I don't know
Headache is an effect of arsenic exposure.	1	yes
	0	no
	999	I don't know
Fever is an effect of arsenic exposure.	1	yes
	0	no
	999	I don't know
Cough is an effect of arsenic exposure.	1	yes
	0	no

	999	I don't know
Cancer is an effect of arsenic exposure.	1	yes
	0	no
	999	I don't know
High blood pressure is an effect of arsenic exposure.	1	yes
	0	no
	999	I don't know
The major health related effects of arsenic exposure can be completely different from day to day.		
	0	no
	999	I don't know
The major effects of arsenic exposure are developped immediately.	1	yes
	0	no
	999	I don't know

The worst major effect of long-term and constant arsenic exposure is cancer on the lungs, the skin and the bladder. There are signs, that arsenic can foster diabetes, can have neurological effects and can cause problems with the heart and blood pressure. These symptoms are together known as "Arsenicosis". Can you please tell me for each of the following whether it is a preventive action to lower arsenic exposure? To drink only clean-looking water.	1	yes
	0	no

	999	I don't know
To let the water have contact with fresh air.	1	yes
	0	no
	999	I don't know
To test the water on arsenic and drink it only if it is classyfied as safe.		
	0	no
	999	I don't know
To boil the water before drinking it.	1	yes
	0	no
	999	I don't know
To drink the water right after collecting and avoid storage of the water.	1	yes
	0	no
	999	I don't know
To collect water in a special bucket.	1	yes
	0	no
	999	I don't know
To avoid microbial	1	yes

contamination of the water.		
	0	no
	999	I don't know
To collect water only from the special filtration unit.	1	yes
	0	no
	999	I don't know
There is no prevention possible for arsenicosis.	1	yes
	0	no
	999	I don't know
To purify the water by adding chlorine	1	yes
	0	no
	999	I don't know
The only thing one can do to prevent from consequences of arsenic exposure is neither to drink water with arsenic pollution nor to cook with it. As well,		

contaminated food crops pose a great threat to one's health. For washing purpose, it doesn't matter whether the water contains arsenic or not.		
How many people of your household do you know that suffer from effects of arsenic exposure?		
How many people outside of your family do you know that suffer from effects of arsenic exposure?		
Compared to persons your sex and age, how much higher or lower are your chances of developing arsenicosis?		
	20	Lower
	3	neither higher nor lower

How much higher?	5	Very much higher
	4	a little higher
How much lower?	2	a little lower
	1	Very much lower
Do you think you are more or less respected by your community because you collect water of the community water scheme?		
	20	less respected
	3	neither more nor less respected
How much more respected do you think you are?	5	very much more
	4	a little more
How much less respected do you think you are?	1	a little less
If you collect water at the community water scheme, to what extent do you think people will envy you?		
	2	A little.

	3	Somewhat.
	4	Very.
	5	Extremely.
Do you feel comfortable or uncomfortable to your community when collecting water at the community water scheme?		
	20	uncomfortable
	3	neither comfortable nor uncomfortable
How comfortable?	4	somewhat comfortable
	5	very much comfortable
How uncomfortable?	2	somewhat uncomfortable
	1	very much uncomfortable
How safe or dangerous is it for you to use the community water scheme for water collection?		
	20	dangerous
	3	neither safe nor dangerous
How safe?	4	somewhat safe
	5	very safe
How dangerous?	2	somewhat dangerous

	1	very dangerous
Introduction: all of the following can be effortful sometimes when fetching water at the community water scheme. What do you think is effortful?		
long distance	1	not effortful at all
	2	a little effortful
	3	rather effortful
	4	effortful
	5	very effortful
spending time	1	not effortful at all
	2	a little effortful
	3	rather effortful
	4	effortful
	5	very effortful
waiting time	1	not effortful at all
	2	a little effortful
	3	rather effortful
	4	effortful
	5	very effortful
walking to the source	1	not effortful at all
	2	a little effortful
	3	rather effortful
	4	effortful

	5	very effortful
pumping / collecting the water	1	not effortful at all
	2	a little effortful
	3	rather effortful
	4	effortful
	5	very effortful
self willingness	1	not effortful at all
	2	a little effortful
	3	rather effortful
	4	effortful
	5	very effortful
keep in mind where to collect the water	1	not effortful at all
	2	a little effortful
	3	rather effortful
	4	effortful
	5	very effortful
be patient	1	not effortful at all
	2	a little effortful
	3	rather effortful
	4	effortful
	5	very effortful
be punctual	1	not effortful at all
	2	a little effortful
	3	rather effortful
	4	effortful

	5	very effortful
planning to collect the water due to the running times of the water scheme	1	not effortful at all
	2	a little effortful
	3	rather effortful
	4	effortful
	5	very effortful
pay for using	1	not effortful at all
	2	a little effortful
	3	rather effortful
	4	effortful
	5	very effortful
share maintenance costs for reparations	1	not effortful at all
	2	a little effortful
	3	rather effortful
	4	effortful
	5	very effortful
maintain water source and piped scheme	1	not effortful at all
	2	a little effortful
	3	rather effortful
	4	effortful
	5	very effortful

maintain good relationship with caretaker	1	not effortful at all
	2	a little effortful
	3	rather effortful
	4	effortful
	5	very effortful
Is there anything else you find effortful at collecting water at the community water scheme? More specifically, how difficult is it to collect enough water as you need from the community water scheme?		
	2	a little difficult
	3	rather difficult
	4	somewhat difficult
	5	very difficult
How sure are you that you can collect as much water as you need at the community water scheme?		

	2	a little sure
	3	rather sure
	4	somewhat sure
	5	very sure
How time-consuming is it to collect water at the community water scheme?	1	not at all
	2	a little
	3	rather
	4	somewhat
	5	very
How difficult is it to find the time and effort to collect your water at the community water scheme?		
	2	a little difficult
	3	rather difficult
	4	somewhat difficult
	5	very difficult
How convenient is it for you to use the community water scheme for water collection?		
	2	a little convenient
	3	rather convenient

	4	somewhat convenient
	5	very convenient
Considering benefits and efforts of collecting water at the community water scheme, how worthwhile do you think it is?		
	2	Not.
	3	A little.
	4	Strongly.
	5	Very.
Multiple! What are positive things of collecting water at the community water scheme?	1	reliable functionality
	2	removes iron
	3	removes salinity
	4	removes arsenic
	5	removes dirt
	6	doesn’t give diarrhea
	7	short distance
	8	time to collect water
	9	physical effort
	10	costs
	11	restriction to collect

	12	meet other people
	13	others see me
	14	other
If other, please specify:		
Multiple! What are negative things of collecting water at the community water scheme?	1	unreliable functionality
	2	iron
	3	salinity
	4	arsenic
	5	dirt
	6	gives diarrhea
	7	distance
	8	time to collect water
	9	physical effort
	10	costs
	11	restriction to collect
	12	meet other people
	13	others see me
	14	other
If other, please specify:		
If you do not collect the water at the community		

water scheme, how do you feel?		
How much do you like the taste of the water collected at the community water scheme?	1	Not at all.
	2	A little.
	3	Somewhat.
	4	Very.
	5	Extremely.
How much do you like the colour of the water collected at the community water scheme?	1	Not at all.
	2	A little.
	3	Somewhat.
	4	Very.
	5	Extremely.
How much do you like the smell of the water collected at the community water scheme?	1	Not at all.
	2	A little.
	3	Somewhat.

	4	Very.
	5	Extremely.
How many members of your community collect water at the community water scheme?	1	almost nobody
	2	some of them
	3	half of them
	4	most of them
	5	everyone
How much of their drinking and cooking water do the members of your community collect from the community water scheme?		
	2	a little bit
	3	half of it
	4	most of it
	5	all of it
How much of their drinking and cooking water do your relatives within your community collect from the		

community water scheme?		
	2	a little bit
	3	half of it
	4	most of it
	5	all of it
How many of your relatives within your community collect water at the community water scheme?		
	2	some of them
	3	half of them
	4	most of them
	5	everyone
You collect water from the community water scheme. Do people who are important to you rather approve or disapprove of this?		
	20	Disapprove
	3	Neither approve nor disapprove
How much do they approve?	4	Somewhat approve

	5	strongly approve
How much do they disapprove?	2	somewhat disapprove
	1	strongly disapprove
You do not collect water from the community water scheme. Do people who are important to you rather approve or disapprove of this?		
	20	Disapprove
	3	Neither approve nor disapprove
How much do they approve?	4	Somewhat approve
	5	strongly approve
How much do they disapprove?	2	somewhat disapprove
	1	strongly disapprove
Imagine you didn't collect drinking water at the communtiy water scheme - how much would people who are important to you approve that you do not collect		

water at the community water scheme?		
	20	Disapprove
	3	Neither approve nor disapprove
How much do they approve?	4	Somewhat approve
	5	strongly approve
How much do they disapprove?	2	somewhat disapprove
	1	strongly disapprove
Imagine you collected drinking water at the communtiy water scheme - how much would people who are important to you approve that you do collect water at the community water scheme?		
	20	Disapprove
	3	Neither approve nor disapprove
How much do they approve?	4	Somewhat approve
	5	strongly approve
How much do they disapprove?	2	somewhat disapprove

	1	strongly disapprove
Who is important to you? People who are leaders in the community (e.g gram panchayat}, how much do they promote that you collect water at the community water scheme?		
	2	A little.
	3	Somewhat.
	4	Very.
	5	Extremely.
How much do religious leaders promote that you collect water at the community water scheme?	1	Not at all.
	2	A little.
	3	Somewhat.
	4	Very.
	5	Extremely.
Multiple! In your opinion, what do the people that are important to		

you dislike about you collecting water at the community water scheme?		
	2	They think the water tastes badly
	3	They think it takes too much time
	4	They think it is too effortful
	5	They think it is shameful
	6	They don't want me to talk to the caretaker
	7	They think it is too expensive
	8	They think the water quality is bad
	9	Other
Multiple! In your opinion, what do these people like about you collecting water at the community water scheme?		
	2	they like the taste of the water
	3	iron free water is important to them
	4	arsenic free water is important to them
	5	they think it is healthy
	6	they like the colour of the water
	7	they like the clear water
	8	other
How strongly do you feel a personal		

obligation to collect all the water at the community water-filter?		
	2	A little.
	3	Somewhat.
	4	Very.
	5	Extremely.
How strongly does the head of household support that you collect water at the water scheme?		
	2	A little.
	3	Somewhat.
	4	Very.
	5	Extremely.
How strongly does the head of household support that you don't collect water at the water scheme?		
	2	A little.
	3	Somewhat.
	4	Very.

	5	Extremely.
How confident are you that you can collect water at the community water scheme even if your relatives continue to consume water from other sources?		
	2	A little.
	3	Somewhat.
	4	Very.
	5	Extremely.
How confident are you that you can collect sufficient water from the community water-filter for your family?		
	2	A little.
	3	Somewhat.
	4	Very.
	5	Extremely.
How confident are you that you can collect drinking water at		

the community water scheme even if the caretaker is rude to you?		
	2	A little.
	3	Somewhat.
	4	Very.
	5	Extremely.
How confident are you that you can continue to collect drinking water all the time, even if problems arise (e.g. some people question your water collection}, at the community water scheme?		
	2	A little.
	3	Somewhat.
	4	Very.
	5	Extremely.
How confident are you that you can always collect your drinking water from the community water		

scheme, even though this may be difficult sometimes (e.g. because it is broken, you have little time to go there}?

2 Not.
3 A little.
4 Strongly.
5 Very.

Imagine that the water collection is not possible at some point at the community water scheme. How confident are you that you will be able to re-start collecting drinking water at the community water scheme again after a couple of days?

2 Not.
3 A little.
4 Strongly.
5 Very.

Imagine that the water collection is not possible during monsoon (rainy season} at the community water scheme. How confident are you that you will be able to re-start collecting drinking water at the community water scheme again after?		
	2	Not.
	3	A little.
	4	Strongly.
	5	Very.
Imagine you occasionally do not go to collect water from the community water scheme. How confident are you about collecting water from the community water-filter regularly again?		

	2	Not.
	3	A little.
	4	Strongly.
	5	Very.
How often does it happen that you want to collect water at the community water scheme but you cannot use it because it is not working?		
	2	Seldom.
	3	From time to time
	4	Frequently
	5	Very often
What is the main reason it is not working?		
How often does it happen that these reasons prevent you from collecting water at the community water scheme?		
Bad relationship with caretaker	1	Never
	2	Seldom.
	3	From time to time

	4	Frequently
	5	Very often
water collection frequency is restricted	1	Never
	2	Seldom.
	3	From time to time
	4	Frequently
	5	Very often
collecting water there is shameful	1	Never
	2	Seldom.
	3	From time to time
	4	Frequently
	5	Very often
going outside the house is bad	1	Never
	2	Seldom.
	3	From time to time
	4	Frequently
	5	Very often
fee for water collection	1	Never
	2	Seldom.
	3	From time to time
	4	Frequently
	5	Very often
water scheme is broken	1	Never
	2	Seldom.

	3	From time to time
	4	Frequently
	5	Very often
long distance	1	Never
	2	Seldom.
	3	From time to time
	4	Frequently
	5	Very often
much time to walk	1	Never
	2	Seldom.
	3	From time to time
	4	Frequently
	5	Very often
long waiting time	1	Never
	2	Seldom.
	3	From time to time
	4	Frequently
	5	Very often
much physical effort	1	Never
	2	Seldom.
	3	From time to time
	4	Frequently
	5	Very often
salinity problem	1	Never
	2	Seldom.
	3	From time to time
	4	Frequently

	5	Very often
microbial contamination	1	Never
	2	Seldom.
	3	From time to time
	4	Frequently
	5	Very often
forgetting to collect water there	1	Never
	2	Seldom.
	3	From time to time
	4	Frequently
	5	Very often
other	1	Never
	2	Seldom.
	3	From time to time
	4	Frequently
	5	Very often
How easy or difficult do you find it to overcome these difficulties? Bad relationship with caretaker	10	difficult
	20	easy
	3	neither difficult nor easy
How difficult?	4	somewhat difficult?
	5	very

How easy?	2	somewhat easy?
	1	very
water collection frequency is restricted	10	difficult
	20	easy
	3	neither difficult nor easy
How difficult?	4	somewhat difficult?
	5	very
How easy?	2	somewhat easy?
	1	very
collecting water there is shameful	10	difficult
	20	easy
	3	neither difficult nor easy
How difficult?	4	somewhat difficult?
	5	very
How easy?	2	somewhat easy?
	1	very
going outside the house is bad	10	difficult
	20	easy
	3	neither difficult nor easy
How difficult?	4	somewhat difficult?
	5	very
How easy?	2	somewhat easy?
	1	very
fee for water collection	10	difficult

	20	easy
	3	neither difficult nor easy
How difficult?	4	somewhat difficult?
	5	very
How easy?	2	somewhat easy?
	1	very
water scheme is broken	10	difficult
	20	easy
	3	neither difficult nor easy
How difficult?	4	somewhat difficult?
	5	very
How easy?	2	somewhat easy?
	1	very
long distance	10	difficult
	20	easy
	3	neither difficult nor easy
How difficult?	4	somewhat difficult?
	5	very
How easy?	2	somewhat easy?
	1	very
much time to walk	10	difficult
	20	easy
	3	neither difficult nor easy
How difficult?	4	somewhat difficult?
	5	very
How easy?	2	somewhat easy?
	1	very

long waiting time	10	difficult
	20	easy
	3	neither difficult nor easy
How difficult?	4	somewhat difficult?
	5	very
How easy?	2	somewhat easy?
	1	very
much physical effort	10	difficult
	20	easy
	3	neither difficult nor easy
How difficult?	4	somewhat difficult?
	5	very
How easy?	2	somewhat easy?
	1	very
salinity problem	10	difficult
	20	easy
	3	neither difficult nor easy
How difficult?	4	somewhat difficult?
	5	very
How easy?	2	somewhat easy?
	1	very
microbial contamination	10	difficult
	20	easy
	3	neither difficult nor easy
How difficult?	4	somewhat difficult?
	5	very
How easy?	2	somewhat easy?

	1	very
forgetting to collect water there	10	difficult
	20	easy
	3	neither difficult nor easy
How difficult?	4	somewhat difficult?
	5	very
How easy?	2	somewhat easy?
	1	very
other	10	difficult
	20	easy
	3	neither difficult nor easy
How difficult?	4	somewhat difficult?
	5	very
How easy?	2	somewhat easy?
	1	very
How often during the last 4 weeks did it happen that you wanted to collect water at the community water scheme but you didn't feel like doing it?		
	2	Seldom.
	3	From time to time
	4	Frequently
	5	Very often

How often during the last 4 weeks did it happen that you wanted to collect water at the community water scheme but you didn’t because you were in a hurry?		
	2	Seldom.
	3	From time to time
	4	Frequently
	5	Very often
How often during the last 4 weeks did it happen that you wanted to collect water at the community water scheme but you didn’t because you thougt it is too effortful?		
	2	Seldom.
	3	From time to time
	4	Frequently
	5	Very often

What are other habits that hinder you? How much do they hinder you to collect water from the community water scheme?	1	Not at all
	2	Not so much
	3	Somewhat
	4	Strongly.
	5	Very.
Who in your household takes the final decision to collect water at the community water scheme?		
Do you have a plan who helps you to collect water from the community based water scheme?		
	2	I disagree a little bit
	3	I neither disagree nor agree
	4	I agree a little bit
	5	I agree very much
Do you have a plan when to		

collect sufficient water for your family for an entire day at the water scheme?		
	2	I disagree a little bit
	3	I neither disagree nor agree
	4	I agree a little bit
	5	I agree very much
Do you have a plan where you can store the water collected at the water scheme safely in your home?		
	2	I disagree a little bit
	3	I neither disagree nor agree
	4	I agree a little bit
	5	I agree very much
Do you have a plan how you can collect water at the community water scheme if the water scheme is not running at that point in time?		
	2	I disagree a little bit
	3	I neither disagree nor agree

	4	I agree a little bit
	5	I agree very much
Do you have a detailed plan what to do when the community water scheme gets broken?		
	2	I disagree a little bit
	3	I neither disagree nor agree
	4	I agree a little bit
	5	I agree very much
Do you have a detailed plan how to avoid forgetting to collect water from the community water scheme?		
	2	I disagree a little bit
	3	I neither disagree nor agree
	4	I agree a little bit
	5	I agree very much
Do you have a detailed plan what to do when other urgent tasks arise and impair my going to collect water		

from the community water schemes?		
	2	I disagree a little bit
	3	I neither disagree nor agree
	4	I agree a little bit
	5	I agree very much
Do you try to comply with your intention to always collect your drinking water at the community water scheme?		
	2	a little true
	3	somewhat true
	4	very true
	5	extremely true
During the last week… … How much did you pay attention so you don't forget to collect water from the community water scheme?		
	2	a little true
	3	somewhat true

	4	very true
	5	extremely true
… How much did you watch yourself to collect sufficient water from the community water scheme for drinking and cooking for your family?		
	2	a little true
	3	somewhat true
	4	very true
	5	extremely true
… How often did you remember your good intentions to collect water from the community water scheme?		
	2	seldom
	3	half of the times
	4	most of the times
	5	everytime
… How aware were you of your goal to collect		

water from the community water scheme?		
	2	a little
	3	rather
	4	very
	5	Extremely.
… How strongly did you try to to collect all of our drinking water from the community water scheme?		
	2	a little
	3	rather
	4	very
	5	Extremely.
… How strongly did you try to make time to collect water from the community water scheme every day?		
	2	a little
	3	rather
	4	very
	5	Extremely.

How often did you forget to collect water from the community water scheme in the last four weeks?		
	2	seldom
	3	half of the times
	4	most of the times
	5	everytime
Multiple! Is there something that helps you to remember to collect water at the community water scheme?		
	2	head of household
	3	other household member
	4	relatives
	5	neighbour
	6	religious leader
	7	empty bucket
	8	before eating
	9	before cooking
	10	seeing the position of the sun
	11	daily routines
	12	others

If other, please specify:		
How much does it bother you when you cannot collect your water at the community water scheme even if you want to for example because the water scheme is not running?		
	2	A little.
	3	Somewhat.
	4	Very.
	5	Extremely.
How important is it for you to collect water from the community water scheme?	1	Not at all.
	2	A little.
	3	Somewhat.
	4	Very.
	5	Extremely.
Do you feel guilty if you don't collect the water		

at the community water scheme?		
	2	A little.
	3	Somewhat.
	4	Very.
	5	Extremely.
How annoyed do you feel if you forget to collect water at the community water scheme?		
	2	A little.
	3	Somewhat.
	4	Very.
	5	Extremely.
How strongly do you intend to collect all your water at the community based water scheme?		
	2	somewhat high intention
	3	rather high intention
	4	quite high intention
	5	very high intention
How strongly do you intend to collect always your water from		

the community water scheme?		
	2	Not.
	3	A little.
	4	Strongly.
	5	Very.
The following questions are related to your water-collection at the taps of the community water schemein your village. Water collection at the water scheme is something…		
… I do frequently.	10	I agree.
	20	I disagree.
	3	I neither agree nor disagree.
How much do you agree?	4	I strongly agree.
	5	I agree a little.
How much do you disagree?	2	I disagree a little.
	1	I strongly disagree.

… I do automatically.	10	I agree.
	20	I disagree.
	3	I neither agree nor disagree.
How much do you agree?	4	I strongly agree.
	5	I agree a little.
How much do you disagree?	2	I disagree a little.
	1	I strongly disagree.
… I do without having to remember actively.	10	I agree.
	20	I disagree.
	3	I neither agree nor disagree.
How much do you agree?	4	I strongly agree.
	5	I agree a little.
How much do you disagree?	2	I disagree a little.
	1	I strongly disagree.
… that makes me feel weird, if I don't collect water there.	10	I agree.
	20	I disagree.
	3	I neither agree nor disagree.
How much do you agree?	4	I strongly agree.

	5	I agree a little.
How much do you disagree?	2	I disagree a little.
	1	I strongly disagree.
… I do without thinking.	10	I agree.
	20	I disagree.
	3	I neither agree nor disagree.
How much do you agree?	4	I strongly agree.
	5	I agree a little.
How much do you disagree?	2	I disagree a little.
	1	I strongly disagree.
… that would require an effort NOT to do.	10	I agree.
	20	I disagree.
	3	I neither agree nor disagree.
How much do you agree?	4	I strongly agree.
	5	I agree a little.
How much do you disagree?	2	I disagree a little.
	1	I strongly disagree.
… that belongs to my daily routine.	10	I agree.
	20	I disagree.
	3	I neither agree nor disagree.

How much do you agree?	4	I strongly agree.
	5	I agree a little.
How much do you disagree?	2	I disagree a little.
	1	I strongly disagree.
… I start doing before I realize I am doing it.	10	I agree.
	20	I disagree.
	3	I neither agree nor disagree.
How much do you agree?	4	I strongly agree.
	5	I agree a little.
How much do you disagree?	2	I disagree a little.
	1	I strongly disagree.
… I would find hard not to collect water there.	10	I agree.
	20	I disagree.
	3	I neither agree nor disagree.
How much do you agree?	4	I strongly agree.
	5	I agree a little.
How much do you disagree?	2	I disagree a little.
	1	I strongly disagree.

… I have no need to think about doing.	10	I agree.
	20	I disagree.
	3	I neither agree nor disagree.
How much do you agree?	4	I strongly agree.
	5	I agree a little.
How much do you disagree?	2	I disagree a little.
	1	I strongly disagree.
… that is typically “me”.	10	I agree.
	20	I disagree.
	3	I neither agree nor disagree.
How much do you agree?	4	I strongly agree.
	5	I agree a little.
How much do you disagree?	2	I disagree a little.
	1	I strongly disagree.
… I have been doing for a long time.	10	I agree.
	20	I disagree.
	3	I neither agree nor disagree.
How much do you agree?	4	I strongly agree.
	5	I agree a little.

How much do you disagree?	2	I disagree a little.
	1	I strongly disagree.
How much do you agree or disagree with the following statement: "Drinking water collection at the community water scheme is a matter of habit for me."		
	20	I disagree.
	3	I neither agree nor disagree.
How much do you agree?	4	I strongly agree.
	5	I agree a little.
How much do you disagree?	2	I disagree a little.
	1	I strongly disagree.
How often do you go to collect water at the water scheme as needed?	1	almost never
	2	seldom
	3	sometimes
	4	often
	5	amost always

How often do you go to collect water at the water scheme before cooking?	1	almost never
	2	seldom
	3	sometimes
	4	often
	5	amost always
How often do you go to collect water at the water scheme after cooking?	1	almost never
	2	seldom
	3	sometimes
	4	often
	5	amost always
How often do you go to collect water at the water scheme before breakfast?	1	almost never
	2	seldom
	3	sometimes
	4	often
	5	amost always
How often do you go to collect water at the	1	almost never

water scheme after waking up?		
	2	seldom
	3	sometimes
	4	often
	5	amost always
How often do you go to collect water at the water scheme before lunch?	1	almost never
	2	seldom
	3	sometimes
	4	often
	5	amost always
How often do you go to collect water at the water scheme in the afternoon?	1	almost never
	2	seldom
	3	sometimes
	4	often
	5	amost always
How often do you go to collect water at the water scheme in the evening?	1	almost never
	2	seldom

	3	sometimes
	4	often
	5	amost always
How often do you go to collect water at the water scheme in the night?	1	almost never
	2	seldom
	3	sometimes
	4	often
	5	amost always
How often do you go to collect water at the water scheme around midday?	1	almost never
	2	seldom
	3	sometimes
	4	often
	5	amost always
How often do you go to collect water at the water scheme in the morning?	1	almost never
	2	seldom
	3	sometimes
	4	often
	5	amost always

How often do you go to collect water at the water scheme when you see the storage is empty?	1	almost never
	2	seldom
	3	sometimes
	4	often
	5	amost always
The following questions are related to your water-collection at your handpump. Water collection at the handpump is something…		
… I do frequently.	10	I agree.
	20	I disagree.
	3	I neither agree nor disagree.
How much do you agree?	4	I strongly agree.
	5	I agree a little.
How much do you disagree?	2	I disagree a little.

	1	I strongly disagree.
… I do automatically.	10	I agree.
	20	I disagree.
	3	I neither agree nor disagree.
How much do you agree?	4	I strongly agree.
	5	I agree a little.
How much do you disagree?	2	I disagree a little.
	1	I strongly disagree.
… I do without having to remember actively.	10	I agree.
	20	I disagree.
	3	I neither agree nor disagree.
How much do you agree?	4	I strongly agree.
	5	I agree a little.
How much do you disagree?	2	I disagree a little.
	1	I strongly disagree.
… that makes me feel weird, if I don't collect water there.		
	20	I disagree.
	3	I neither agree nor disagree.

How much do you agree?	4	I strongly agree.
	5	I agree a little.
How much do you disagree?	2	I disagree a little.
	1	I strongly disagree.
… I do without thinking.	10	I agree.
	20	I disagree.
	3	I neither agree nor disagree.
How much do you agree?	4	I strongly agree.
	5	I agree a little.
How much do you disagree?	2	I disagree a little.
	1	I strongly disagree.
… that would require an effort NOT to do.		
	20	I disagree.
	3	I neither agree nor disagree.
How much do you agree?	4	I strongly agree.
	5	I agree a little.
How much do you disagree?	2	I disagree a little.
	1	I strongly disagree.
… that belongs to my daily routine.	10	I agree.
	20	I disagree.

	3	I neither agree nor disagree.
How much do you agree?	4	I strongly agree.
	5	I agree a little.
How much do you disagree?	2	I disagree a little.
	1	I strongly disagree.
… I start doing before I realize I am doing it.		
	20	I disagree.
	3	I neither agree nor disagree.
How much do you agree?	4	I strongly agree.
	5	I agree a little.
How much do you disagree?	2	I disagree a little.
	1	I strongly disagree.
… I would find hard not to collect water there.		
	20	I disagree.
	3	I neither agree nor disagree.
How much do you agree?	4	I strongly agree.
	5	I agree a little.
How much do you disagree?	2	I disagree a little.
	1	I strongly disagree.

… I have no need to think about doing.		
	20	I disagree.
	3	I neither agree nor disagree.
How much do you agree?	4	I strongly agree.
	5	I agree a little.
How much do you disagree?	2	I disagree a little.
	1	I strongly disagree.
… that is typically “me”.	10	I agree.
	20	I disagree.
	3	I neither agree nor disagree.
How much do you agree?	4	I strongly agree.
	5	I agree a little.
How much do you disagree?	2	I disagree a little.
	1	I strongly disagree.
… I have been doing for a long time.	10	I agree.
	20	I disagree.
	3	I neither agree nor disagree.
How much do you agree?	4	I strongly agree.
	5	I agree a little.

How much do you disagree?	2	I disagree a little.
	1	I strongly disagree.
Do you agree or disagree with the following statement: "Water collection at the handpump is a matter of habit for me."		
	20	I disagree.
	3	I neither agree nor disagree.
How much do you agree?	4	I strongly agree.
	5	I agree a little.
How much do you disagree?	2	I disagree a little.
	1	I strongly disagree.
How often do you go to collect water at the handpump as needed?	1	almost never
	2	seldom
	3	sometimes
	4	often
	5	amost always
How often do you go to collect water at the	1	almost never

handpump before cooking?		
	2	seldom
	3	sometimes
	4	often
	5	amost always
How often do you go to collect water at the handpump after cooking?	1	almost never
	2	seldom
	3	sometimes
	4	often
	5	amost always
How often do you go to collect water at the handpump before breakfast?	1	almost never
	2	seldom
	3	sometimes
	4	often
	5	amost always
How often do you go to collect water at the handpump after waking up?	1	almost never
	2	seldom

	3	sometimes
	4	often
	5	amost always
How often do you go to collect water at the handpump before lunch?	1	almost never
	2	seldom
	3	sometimes
	4	often
	5	amost always
How often do you go to collect water at the handpump in the afternoon?	1	almost never
	2	seldom
	3	sometimes
	4	often
	5	amost always
How often do you go to collect water at the handpump in the evening?	1	almost never
	2	seldom
	3	sometimes
	4	often
	5	amost always

How often do you go to collect water at the handpump in the night?	1	almost never
	2	seldom
	3	sometimes
	4	often
	5	amost always
How often do you go to collect water at the handpump around midday?	1	almost never
	2	seldom
	3	sometimes
	4	often
	5	amost always
How often do you go to collect water at the handpump in the morning?	1	almost never
	2	seldom
	3	sometimes
	4	often
	5	amost always
How often do you go to collect water at the	1	almost never

handpump when you see the storage is empty?		
	2	seldom
	3	sometimes
	4	often
	5	amost always

Supplementary materials S4:

Fit indices of three dimensional psychological ownership scale

The three-dimensional measurement scale showed acceptable fit indices: a significant chi-square (χ 2 = 187.699, 77 d.f., p < .001), low RMSEA = 0.077 [.063; .091], and moderate CFI = 0.865. Completely standardized factor loadings ranged from 0.305 to 0.725.

After one step of modifications, the shortened three-dimensional measurement scale showed good fit indices: a significant chi-square (χ 2 = 67.419, 27 d.f., p < .001), low RMSEA = 0.082 [.058; .106], and moderate to high CFI = 0.93. Completely standardized factor loadings ranged from 0.454 to 0.769.
